# Safety profile of small interfering RNA therapeutics: a systematic review and meta-analysis of randomized controlled trials

**DOI:** 10.3389/fdsfr.2026.1855572

**Published:** 2026-07-10

**Authors:** Cheewon Ahn, Hyunsuk Jeong, Hyeon Woo Yim, Na Jin Kim, Sungpil Han, Ilhoan Oh

**Affiliations:** 1 Department of Preventive Medicine, College of Medicine, The Catholic University of Korea, Seoul, Republic of Korea; 2 Medical Library, The Catholic University of Korea, Seoul, Republic of Korea; 3 Department of Pharmacology, College of Medicine, The Catholic University of Korea, Seoul, Republic of Korea; 4 Pharmacometrics Institute for Practical Education and Training (PIPET), College of Medicine, The Catholic University of Korea, Seoul, Republic of Korea; 5 Catholic High-Performance Cell Therapy Center and Department of Medical Life Science, College of Medicine, The Catholic University of Korea, Seoul, Republic of Korea

**Keywords:** clinical trials, meta-analysis, safety, small interfering RNAs, systematic review

## Abstract

**Objectives:**

Small interfering RNAs (siRNAs) represent a promising therapeutic platform addressing unmet clinical needs. Recent advances have improved siRNA design, stability, specificity, and delivery; however, translating preclinical findings into clinical practice remains challenging due to the lack of standardized safety evaluation frameworks. This study conducted a systematic review and meta-analysis of placebo-controlled randomized clinical trials (RCTs) to quantitatively assess the safety of siRNA therapeutics and evaluate the adequacy of current preclinical safety assessments.

**Methods:**

PubMed, Embase, and Cochrane databases were searched from inception to July 2025. Two reviewers independently performed study selection and data extraction according to predefined criteria. Safety outcomes included death, serious adverse events (SAEs), severe adverse events, and adverse events (AEs) leading to treatment discontinuation. Pooled relative risks (RRs) and 95% confidence intervals (CIs) were calculated using R packages *meta* and *metafor*. Methodological quality for AE assessment was evaluated using the McHarm scale. The protocol was registered with PROSPERO (CRD420251120555).

**Results:**

Of 5,527 records screened, 57 studies (53 reports) evaluating 28 siRNA agents were included. Compared with controls, siRNA treatment did not increase the risk of death (RR 0.77, 95% CI 0.62–0.95) and severe AEs (RR 0.85, 95% CI 0.76–0.95), with no statistical heterogeneity (I^2^ = 0%). Risks of SAEs (RR 0.94, 95% CI 0.88–1.00) and treatment discontinuation (RR 0.86, 95% CI 0.66–1.13) were not increased.

**Conclusion:**

Chemical and delivery advancements have effectively enhanced siRNA safety. Nevertheless, ongoing vigilance and comprehensive safety monitoring during early clinical phases remain essential to identify unforeseen toxicities.

**Systematic Review Registration:**

Identifier CRD420251120555.

## Introduction

The management of rare hereditary diseases has largely been confined to symptomatic management rather than curative treatments, resulting in unmet clinical needs for patients (Chaud et al., 2025). To address this limitation, oligonucleotide (OGN)-based therapeutics have emerged as a promising strategy (Levin, 2017). Small interfering RNAs (siRNAs) are a powerful tool for modulating gene expression in a sequence-specific manner (Ranjbar et al., 2023). The mechanism of siRNA involves the binding of its guide strand to the RNA-induced silencing complex (RISC), which triggers RNA interference (RNAi) within the cell to achieve a gene-silencing effect (Chi et al., 2017). The identification of this interference process led to development of agents that target disease-causing genes in rare genetic disorders as well as several common (Ranjbar et al., 2023; Wang et al., 2020). To date, seven siRNA therapeutics have received FDA approval (inclisiran, lumasiran, givosiran, patisiran, vutrisiran, nedosiran, and fitusiran), with several others currently undergoing evaluation for efficacy and safety in clinical trials.

Early siRNA therapeutic agents showed numerous limitations, including low *in vivo* stability leading to rapid degradation, as well as potential toxicity issues such as off-target effects and immunogenicity (Alshaer et al., 2021). The currently approved siRNA therapies and those in development have incorporated several chemical modifications to overcome these problems (Khvorova and Watts, 2017). Key modifications include a phosphorothioate (PS) backbone and ribose modifications like 2′-O-methyl (2′-OME) and 2′-fluoro (2′-F), which enhance resistance to nuclease degradation and reduce immunogenicity (Judge et al., 2006; Roberts et al., 2020). To improve intracellular delivery efficiency, triantennary N-acetylgalactosamine (GalNAc) conjugation was introduced (Springer and Dowdy). GalNAc-conjugates target the asialoglycoprotein receptor (ASGPR), which is predominantly expressed on hepatocytes, facilitating uptake via receptor-mediated endocytosis (Springer and Dowdy; Nair et al., 2014). This mechanism enables highly selective delivery to the liver and minimizes unnecessary drug distribution to other tissues (Janas et al., 2018). Owing to these advantages, GalNAc has become the preferred drug delivery system for liver-targeting siRNA therapeutics (Anand et al., 2025). In silico analysis has also been used to design sequences that minimize off-target effects (Goyenvalle et al., 2023; Patzel, 2007) and preclinical safety assessments have been performed to predict and mitigate potential toxicity before advancing siRNA agents to clinical trials (Goyenvalle et al., 2023; Andersson, 2022). These toxicology studies, performed in compliance with Good Laboratory Practices (GLP), determines the initial starting dose for human clinical trials (Jeon et al., 2022).

Despite these advancements, translating preclinical findings to the clinical setting has been challenging because of the absence of a gold-standard guideline or regulation for the safety assessment of siRNA therapeutics (Ranjbar et al., 2023). While the FDA released a draft guidance on preclinical safety studies for oligonucleotide drugs in November 2024 (USFaD, 2024), whether this guidance adequately addresses the unique characteristics of siRNA therapies has not been determined. This suggests that safety issues not identified in the preclinical phase could potentially be observed as adverse events in clinical trials. Determining whether reported safety issues are attributable to siRNA therapeutics can be challenging, as the targeted diseases are often intractable, with high baseline mortality and hospitalization rates. Therefore, to properly evaluate the safety profile of these agents, comparative studies against a placebo or treatment-as-usual (TAU) with a randomized-controlled design, rather than single-arm studies, are necessary. While individual randomized controlled trials (RCTs) reporting the efficacy and safety of siRNA therapeutics have been published, a systematic review and meta-analysis synthesizing this safety data is currently lacking. Thus, we conducted a systematic review and meta-analysis of published placebo-controlled RCTs to quantitatively assess the safety of siRNA therapeutics that have entered clinical development. Through this analysis, we evaluated the adequacy of the current preclinical safety framework for siRNA-based drugs.

## Methods

### Study registration

The protocol for this systematic review was registered with the International Prospective Register of Systematic Reviews (PROSPERO) (CRD420251120555).

### Search strategy and study selection

We conducted a systematic search and critical review of the literature published from inception through July 2025 in MEDLINE, Embase, and Cochrane databases following the Preferred Reporting Items for Systematic Reviews and Meta-Analyses (PRISMA) guidelines (http://www.prisma-statement.org/) (Supplementary Materials S1, S2). Eligibility criteria were as follows: (Chaud et al., 2025) RCTs that used siRNA drugs as the intervention; and (Levin, 2017) studies that used placebo or treatment-as-usual group as comparison. Exclusion criteria were as follows: 1) reviews, commentaries, or editorials; 2) studies published in abstract or poster presentations; 3) open-label single arm studies; 4) Phase 1 trials with healthy volunteers only; 5) pharmacokinetics or pharmacodynamics studies; and 6) *in vitro* or *in vivo* studies. Study selection and data extraction were conducted independently by two reviewers (CA, HJ) following these pre-specified criteria. The two investigators independently screened all titles and abstracts to extract relevant studies that met the inclusion and exclusion criteria and then independently assessed a full text review for the relevant studies. Disagreements were resolved by consensus involving a third reviewer (HWY).

### Data extraction

Two investigators extracted data using customized data extraction forms. The following data were extracted: year of publication, drug name, indication for use, disease category, number of participants, follow-up period, delivery mechanism, chemical modification, trial phase, and development status. In the disease category, an indication was classified as “rare” if it had a low prevalence (affecting fewer than 200,000 Americans in the US) and no curative treatment was available. An indication was classified as “common” if the prevalence rate was high (affecting more than 200,000 Americans in the US) or standard treatment option was available.

Safety outcomes, including death, serious adverse events (SAEs), severe adverse events, and adverse events (AEs) leading to treatment discontinuation, were extracted from each study. For the intervention group, data on the number of participants who received at least one dose of siRNA and the corresponding number of events for each safety outcome were collected. For the control group, data on the total number of participants randomized and the number of events for each safety outcome were also obtained.

### Quality assessment

Methodological quality of AE assessment was evaluated using questions from the McHarm quality assessment scale for AEs. Reports were evaluated as to whether studies used precise, pre-defined criteria for classifying AEs (e.g., “death,” “serious adverse event”), specified personnel and methods involved in harms collection, and provided quantitative data for both the total number of adverse events and the number of participants affected. For assessing mode of harms collection, active collection was defined as proactive solicitation of adverse event data by an investigator or qualified designee using methods such as checklists or interviews. Passive collection was defined as unsolicited, spontaneous reports of adverse events initiated by study participants. Each item was scored as a “yes,” “no,” or “unable to determine” (Chou et al., 2010). Assessment was based on the main publication, and the trial protocol was also referenced when available. Each item was rated “yes” if specified in the publication or protocol or “no” if not specified. Items for which a definitive assessment could not be made because of an inaccessible protocol were marked as “unable to determine.” The McHarm score for each study was calculated as the total number of items rated “yes.” The McHarm score ranges from 0 to 15, with higher scores indicating a higher quality of harm reporting. Quality assessments were independently conducted by two authors (CA and HJ). Disagreements were resolved by discussion with a third author (HWY).

### Statistical analyses

We estimated the pooled effect using either Mantel-Haenszel method for the fixed effect model or DerSimonian-Laird method for the random-effect model. We presented forest plots in the safety outcomes including death, SAEs, severe adverse events, and AEs leading to treatment discontinuation as relative risks (RRs) and 95% confidence intervals (CIs) for the intervention group compared with the control group. For studies reporting zero events in one or both arms, a standard continuity correction of 0.5 was added to all cells to enable the calculation of relative risks for rare events. An RR less than 1 was interpreted as indicating fewer occurrences of death, SAEs, severe adverse events, and AE leading to treatment discontinuation in the intervention group compared with the control group. Statistical heterogeneity across the studies was assessed using the I^2^ statistics and defined as low (25%–50%), moderate (50%–75%), or high (>75%). Subgroup analyses were conducted to explore whether the risk of death varied by disease category, delivery mechanism, study phase, and product development status. Disease categories were classified as either rare or common. For the delivery mechanism, because of the predominance of studies using GalNAc conjugation, subgroups were defined as GalNAc-based versus non-GalNAc delivery. The study phase was categorized as Phase 1, Phase 2, or Phase 3. Product development status was classified into commercially available products (marketed) versus those still in development or discontinued/terminated. For each subgroup, the number of included trials and the number of participants in both the intervention and control groups were represented. For each subgroup, the effect estimates for the risk of death in the intervention group compared with the control group were presented as RRs with corresponding 95% CIs. Publication bias was assessed using funnel plots, and asymmetry in the plots was examined to evaluate the presence of potential publication bias. All analyzes used the R “meta” and “metafor” packages.

## Results

### Literature search

**FIGURE 1:**
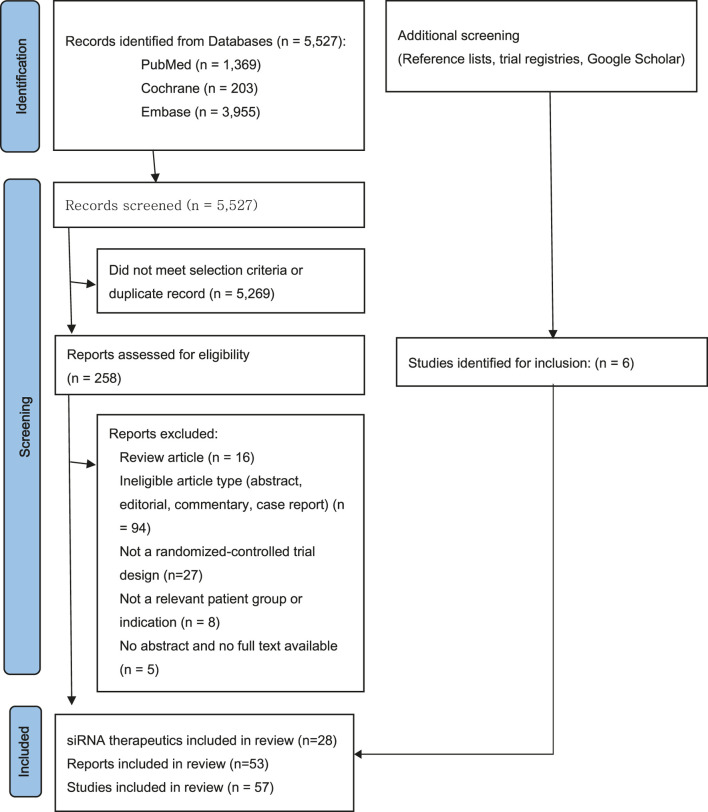
PRISMA 2020 flow diagram of the literature search process for identifying studies.

The initial database search identified 5,527 articles. After removing duplicates and screening titles and abstracts, 258 articles underwent a full-text review for eligibility. During the full-text review, 94 articles were excluded for being an ineligible article type (e.g., abstracts only) and 53 were excluded for being trial protocols or containing only trial results. An additional 27 reports were excluded for not having a randomized controlled trial design, and 8 were excluded for enrolling only healthy volunteers. Through this process, 47 reports were initially selected. A subsequent manual search of the reference lists of included articles and a search of clinicaltrials.gov for registered siRNA trials identified an additional 6 reports. Finally, a total of 53 reports were included Nissen et al., 2023; Nissen et al., 2025; O’Donoghue et al., 2022; Gane E. J. et al., 2023; Benitez-Del-Castillo et al., 2016; Nissen et al., 2024a; Nissen et al., 2024b; Bakris et al., 2024; Desai et al., 2023; Rosenson et al., 2024; Desai et al., 2025; Koren et al., 2022; Nissen et al., 2022; Gottlieb et al., 2016; Gane E. et al., 2023; Ray et al., 2025a; Ray et al., 2025b; Fabbrini et al., 2024; Zamora et al., 2011; Suzuki et al., 2017; Yuen et al., 2020; Hou et al., 2024, Raal et al., 2023; Raal et al., 2020; Ray et al., 2017; Ray et al., 2020; Huo et al., 2024; Yamashita et al., 2024; Watts et al., 2025; Luo et al., 2023; Ballantyne et al., 2024; Gaudet et al., 2024; Wiegman et al., 2025; Koren et al., 2024; Taub et al., 2025; Nissen et al., 2024a; Nissen et al., 2024b; Bakris et al., 2024; Desai et al., 2023; Rosenson et al., 2024; Desai et al., 2025; Koren et al., 2022; Nissen et al., 2022; Gottlieb et al., 2016; Gane E. et al., 2023; Ray et al., 2025a; Ray et al., 2025b; Fabbrini et al., 2024; Zamora et al., 2011; Suzuki et al., 2017; Yuen et al., 2020; Hou et al., 2024; Koren et al., 2024; Taub et al., 2025. Among this group, 4 reports each presented 2 separate studies (Ray et al., 2020; Benitez-Del-Castillo et al., 2016; Fabbrini et al., 2024; Yuen et al., 2020); thus, 57 studies from 53 reports covering 28 different siRNA agents were included in the analysis (Figure 1).

### Study characteristics

The included RCTs were published from 2011 onwards, with 33 studies (58%) published in 2023 or later. Out of 28 distinct siRNA therapeutic agents, 12 (43%) were developed exclusively for rare diseases (Adams et al., 2018; Coelho et al., 2013; Frishberg et al., 2021; Garrelfs et al., 2021; Balwani et al., 2020; Maurer et al., 2023; Sardh et al., 2019; Turner et al., 2018; Barratt et al., 2024; Clark et al., 2024; Baum et al., 2023; Goldfarb et al., 2023; Fontana et al., 2025; Judge et al., 2020; Srivast et al., 2023; Young et al., 2023; Doi et al., 2023), 14 (50%) for common diseases (Thielmann et al., 2021; Nissen et al., 2023; Nissen et al., 2025; O'Donoghue et al., 2022; Gane E. J. et al., 2023; Benitez-Del-Castillo et al., 2016; Nissen et al., 2024a; Nissen et al., 2024b; Bakris et al., 2024; Desai et al., 2023; Rosenson et al., 2024; Desai et al., 2025; Koren et al., 2022; Nissen et al., 2022; Gottlieb et al., 2016; Gane E. et al., 2023; Ray et al., 2025a; Ray et al., 2025b; Fabbrini et al., 2024; Zamora et al., 2011; Suzuki et al., 2017; Yuen et al., 2020; Hou et al., 2024), and 2 (7%) for indications spanning both categories (Raal et al., 2023; Raal et al., 2020; Ray et al., 2017; Ray et al., 2020; Huo et al., 2024; Yamashita et al., 2024; Watts et al., 2025; Luo et al., 2023; Ballantyne et al., 2024; Gaudet et al., 2024; Wiegman et al., 2025; Koren et al., 2024; Taub et al., 2025). Of the 57 studies, 37 focused on common diseases (Raal et al., 2020; Ray et al., 2017; Ray et al., 2020; Thielmann et al., 2021; Huo et al., 2024; Yamashita et al., 2024; Luo et al., 2023; Ballantyne et al., 2024; Gaudet et al., 2024; Nissen et al., 2023; Nissen et al., 2025; O'Donoghue et al., 2022; Gane E. J. et al., 2023; Benitez-Del-Castillo et al., 2016; Nissen et al., 2024a; Nissen et al., 2024b; Bakris et al., 2024; Desai et al., 2023; Rosenson et al., 2024; Desai et al., 2025; Koren et al., 2022; Nissen et al., 2022; Gottlieb et al., 2016; Gane E. et al., 2023; Ray et al., 2025a; Ray et al., 2025b; Fabbrini et al., 2024; Zamora et al., 2011; Suzuki et al., 2017; Yuen et al., 2020; Hou et al., 2024; Koren et al., 2024; Taub et al., 2025), and hyperlipidemia was the most frequently investigated condition (21 studies) (Raal et al., 2020; Ray et al., 2017; Ray et al., 2020; Huo et al., 2024; Yamashita et al., 2024; Luo et al., 2023; Ballantyne et al., 2024; Gaudet et al., 2024; Nissen et al., 2023; Nissen et al., 2025; O'Donoghue et al., 2022; Nissen et al., 2024a; Nissen et al., 2024b; Rosenson et al., 2024; Koren et al., 2022; Nissen et al., 2022; Ray et al., 2025a; Ray et al., 2025b; Koren et al., 2024; Taub et al., 2025). The distribution by clinical trial phase was as follows: 16 Phase 1 (28%) (Coelho et al., 2013; Sardh et al., 2019; Luo et al., 2023; Turner et al., 2018; Nissen et al., 2023; Goldfarb et al., 2023; Gane E. J. et al., 2023; Nissen et al., 2024a; Desai et al., 2023; Koren et al., 2022; Nissen et al., 2022; Ray et al., 2025a; Fabbrini et al., 2024; Suzuki et al., 2017; Doi et al., 2023), 24 Phase 2 (42%) (Frishberg et al., 2021; Ray et al., 2017; Thielmann et al., 2021; Yamashita et al., 2024; Ballantyne et al., 2024; Gaudet et al., 2024; Barratt et al., 2024; Clark et al., 2024; Nissen et al., 2025; Baum et al., 2023; O'Donoghue et al., 2022; Benitez-Del-Castillo et al., 2016; Nissen et al., 2024b; Bakris et al., 2024; Rosenson et al., 2024; Desai et al., 2025; Gottlieb et al., 2016; Gane E. et al., 2023; Ray et al., 2025b; Zamora et al., 2011; Yuen et al., 2020; Hou et al., 2024), and 17 Phase 3 (30%) (Adams et al., 2018; Garrelfs et al., 2021; Balwani et al., 2020; Maurer et al., 2023; Raal et al., 2023; Raal et al., 2020; Ray et al., 2020; Huo et al., 2024; Watts et al., 2025; Fontana et al., 2025; Judge et al., 2020; Wiegman et al., 2025; Srivast et al., 2023; Young et al., 2023; Koren et al., 2024; Taub et al., 2025) studies. A total of 10 studies involved discontinuation of drug development or early termination of clinical trial (Thielmann et al., 2021; Turner et al., 2018; Gane E. J. et al., 2023; Judge et al., 2020; Gottlieb et al., 2016; Zamora et al., 2011; Yuen et al., 2020; Hou et al., 2024); in 4 of these cases, discontinuation was because of safety concerns related to the siRNA agent (Turner et al., 2018; Judge et al., 2020; Yuen et al., 2020). Regarding chemical modifications, 6 of the 7 FDA-approved therapeutics used GalNAc conjugation along with 2′-OMe, 2′-F, and PS backbone modifications (Frishberg et al., 2021; Garrelfs et al., 2021; Balwani et al., 2020; Raal et al., 2023; Raal et al., 2020; Ray et al., 2017; Ray et al., 2020; Sardh et al., 2019; Huo et al., 2024; Yamashita et al., 2024; Luo et al., 2023; Baum et al., 2023; Goldfarb et al., 2023; Fontana et al., 2025; Wiegman et al., 2025; Srivast et al., 2023; Young et al., 2023; Koren et al., 2024; Taub et al., 2025). Therapeutic agents currently in development also feature these modifications, with 4 agents incorporating other variations (Benitez-Del-Castillo et al., 2016; Gane E. et al., 2023; Suzuki et al., 2017; Doi et al., 2023). The number of participants in the 57 RCTs ranged from 6 to 1,617, and the follow-up duration ranged from 10 days (0.3 months) to a maximum of 36 months (Table 1).

**TABLE 1:** Baseline characteristics of the 57 included trials.

First author, year [References]	Drug name	Indication	Disease category	Sample size (Tx; co)	Follow up (mo)	Delivery mechanism	Chemical modification	Trial phase	Development status[trial number]
Adams et al. (2018)	Patisiran	hARRT amyloidosis	Rare	148; 77	18	LNP	2′-OMe	3	Approved (ONPATTRO) [NCT01960348]
Coelho et al. (2013)	ALN-TTR01	hARRT amyloidosis	Rare	24; 8	1	LNP	2′-OMe	1	Discontinued [NCT01148953]
Frishberg et al. (2021)	Lumasiran	PH1	Rare	17; 3	18	GalNAc	2′-OMe, 2′-F, PS	2	Approved (OXLUMO) [NCT02706886]
Garrelfs et al. (2021)	Lumasiran	PH1	Rare	26; 13	6	GalNAc	2′-OMe, 2′-F, PS	3	Approved (OXLUMO) [NCT03681184]
Balwani et al. (2020)	Givosiran	Acute hepatic porphyria	Rare	48; 46	6	GalNAc	2′-OMe, 2′-F, PS	3	Approved (GIVLAARI) [NCT03338816]
Maurer et al. (2023)	Patisiran	hARRT amyloidosis	Rare	181; 179	12	LNP	2′-OMe	3	Approved (ONPATTRO) [NCT03997383]
Raal et al. (2023)	Inclisiran	HoFH	Rare	37; 19	5	GalNAc	2′-OMe, 2′-F, PS	3	Approved (LEQVIO) [NCT03851705]
Raal et al. (2020)	Inclisiran	HeFH or ASCVD	Common	242; 240	18	GalNAc	2′-OMe, 2′-F, PS	3	Approved (LEQVIO) [NCT03397121]
Ray et al. (2017)	Inclisiran	HeFH or ASCVD	Common	370; 127	7	GalNAc	2′-OMe, 2′-F, PS	2	Approved (LEQVIO) [NCT02597127]
Ray et al. (2020)*	Inclisiran	HeFH or ASCVD	Common	781; 780	18	GalNAc	2′-OMe, 2′-F, PS	3	Approved (LEQVIO) [NCT03399370]
Ray et al. (2020)*	Inclisiran	HeFH or ASCVD	Common	810; 807	18	GalNAc	2′-OMe, 2′-F, PS	3	Approved (LEQVIO) [NCT03400800]
Sardh et al. (2019)	Givosiran	Acute hepatic porphyria	Rare	33; 10	3	GalNAc	2′-OMe, 2′-F, PS	1	Approved (GIVLAARI) [NCT02452372]
Thielmann et al. (2021)	Teprasiran	AKI	Common	165; 176	3	Unmodified	2′-OMe	2	Terminated [NCT02610283]
Huo et al. (2024)	Inclisiran	HeFH or ASCVD	Common	171; 174	12	GalNAc	2′-OMe, 2′-F, PS	3	Approved (LEQVIO) [NCT04765657]
Yamashita et al. (2024)	Inclisiran	HeFH or ASCVD	Common	255; 57	12	GalNAc	2′-OMe, 2′-F, PS	2	Approved (LEQVIO) [NCT04666298]
Watts et al. (2025)	Plozasiran	FCS or symptomatic persistent chylomicronemia	Rare	50; 25	12	GalNAc	2′-OMe, 2′-F, PS	3	Ongoing [NCT05089084]
Luo et al. (2023)	Inclisiran	Elevated LDL-C	Common	30; 10	3	GalNAc	2′-OMe, 2′-F, PS	1	Approved (LEQVIO) [NCT04774003]
Ballantyne et al. (2024)	Plozasiran	Mixed hyperlipidemia	Common	266; 87	12	GalNAc	2′-OMe, 2′-F, PS	2	Ongoing [NCT04998201]
Gaudet et al. (2024)	Plozasiran	Severe hypertriglyceridemia	Common	166; 60	12	GalNAc	2′-OMe, 2′-F, PS	2	Ongoing [NCT04720534]
Turner et al. (2018)	ARC-AAT	Alpha-1 antitrypsin deficiency	Rare	7; 4	1	ARC-EX1	2′-OMe, 2′-F, PS	1	Terminated [NCT02363946]
Barratt et al. (2024)	Cemdisiran	IgA nephropathy	Rare	22; 9	9	GalNAc	2′-OMe, 2′-F, PS	2	Ongoing [NCT03841448]
Clark et al. (2024)	Fazirsiran	Alpha-1 antitrypsin deficiency	Rare	26; 14	22	GalNAc	2′-OMe, 2′-F, PS	2	Ongoing [NCT03945292]
Nissen et al. (2023)	Lepodisiran	Lipoprotein disorder	Common	36; 12	12	GalNAc	2′-OMe, 2′-F, PS	1	Ongoing [NCT04914546]
Nissen et al. (2025)	Lepodisiran	Lipoprotein disorder	Common	251; 69	18	GalNAc	2′-OMe, 2′-F, PS	2	Ongoing [NCT05565742]
Baum et al. (2023)	Nedosiran	PH1 or PH2	Rare	23; 12	6	GalNAc	2′-OMe, 2′-F, PS	2	Approved (Rivfloza) [NCT03847909]
Goldfarb et al. (2023)	Nedosiran	PH3	Rare	4; 2	3	GalNAc	2′-OMe, 2′-F, PS	1	Approved (Rivfloza) [NCT04555486]
O'Donoghue et al. (2022)	Olpasiran	ASCVD	Common	227; 54	18	GalNAc	2′-OMe, 2′-F, PS	2	Ongoing [NCT04270760]
Gane E. J. et al. (2023)	RG6346	Chronic HBV infection	Common	18; 9	4	GalNAc	2′-OMe, 2′-F, PS	1	Discontinued [NCT03772249]
Benitez-Del-Castillo et al. (2016) ^†^	SYL1001	Dry eye disease	Common	40; 20	0.6	Unmodified	Unmodified	2	Ongoing [NCT01776658]
Benitez-Del-Castillo et al. (2016) ^†^	SYL1001	Dry eye disease	Common	44; 24	0.6	Unmodified	Unmodified	2	Ongoing [NCT02455999]
Fontana et al. (2025)	Vutrisiran	ATTR-CM	Rare	326; 328	36	GalNAc	2′-OMe, 2′-F, PS	3	Approved (AMVUTTRA) [NCT04153149]
Nissen et al. (2024a)	Zerlasiran	ASCVD	Common	27; 9	6	GalNAc	2′-OMe, 2′-F, PS	1	Ongoing [NCT04606602]
Nissen et al. (2024b)	Zerlasiran	ASCVD	Common	131; 47	15	GalNAc	2′-OMe, 2′-F, PS	2	Ongoing [NCT05537571]
Bakris et al. (2024)	Zilebesiran	Hypertension	Common	302; 75	6	GalNAc	2′-OMe, 2′-F, PS	2	Ongoing [NCT04936035]
Desai et al. (2023)	Zilebesiran	Hypertension	Common	64; 32	6	GalNAc	2′-OMe, 2′-F, PS	1	Ongoing [NCT03934307]
Rosenson et al. (2024)	Zodasiran	Mixed hyperlipidemia	Common	153; 51	6	GalNAc	2′-OMe, 2′-F, PS	2	Ongoing [NCT04832971]
Judge et al. (2020)	Revusiran	hATTR-CM	Rare	140; 66	6	GalNAc	2′-OMe, 2′-F, PS	3	Discontinued [NCT02319005]
Desai et al. (2025)	Zilebesiran	Hypertension	Common	329; 329	6	GalNAc	2′-OMe, 2′-F, PS	2	Ongoing [NCT05103332]
Koren et al. (2022)	Olpasiran	Elevated LP(a)	Common	48; 16	12	GalNAc	2′-OMe, 2′-F, PS	1	Ongoing [NCT03626662]
Nissen et al. (2022)	Zerlasiran	Elevated LP(a)	Common	24; 8	6	GalNAc	2′-OMe, 2′-F, PS	1	Ongoing [NCT04606602]
Wiegman et al. (2025)	Inclisiran	HoFH	Rare	9; 4	12	GalNAc	2′-OMe, 2′-F, PS	3	Approved (LEQVIO) [NCT04659863]
Gottlieb et al. (2016)	ALN-RSV01	BOS by RSV infection in LTx recipients	Common	45; 42	1	Unmodified	Unmodified	2	Discontinued [NCT01065935]
Gane E. J. et al. (2023)	VIR-2218	Chronic HBV infection	Common	24; 8	12	GalNAc	2′-O-MOE, 2′-F, S-GNA, PS	2	Ongoing [NCT03672188]
Ray et al. (2025a)	Solbinsiran	Mixed hyperlipidemia	Common	42; 14	6	GalNAc	2′-OMe, 2′-F, PS	1	Ongoing [NCT04644809]
Ray et al. (2025a)	Solbinsiran	Mixed hyperlipidemia	Common	147; 57	9	GalNAc	2′-OMe, 2′-F, PS	2	Ongoing [NCT05256654]
Fabbrini et al., 2024 ^‡^	JNJ-75220795	MAFLD	Common	6; 3	6	GalNAc	2′-OMe, 2′-F, PS	1	Ongoing [NCT05039710]
Fabbrini et al., 2024 ^‡^	JNJ-75220795	MAFLD	Common	39; 16	6	GalNAc	2′-OMe, 2′-F, PS	1	Ongoing [NCT04844450]
Zamora et al. (2011)	ALN-RSV01	BOS by RSV infection	Common	16; 8	1	Unmodified	Unmodified	2	Discontinued [NCT00658086]
Suzuki et al. (2017)	STNM01	Chron’s disease	Common	9; 9	1	Undisclosed	Undisclosed	1	Ongoing
Yuen et al., 2020 ^§^	ARC-520	Chronic HBV infection	Common	38; 20	6	ARC-EX1	2′-OMe, 2′-F, PS	2	Terminated [NCT02604199]
Yuen et al., 2020 ^§^	ARC-520	Chronic HBV infection	Common	21; 11	6	ARC-EX1	2′-OMe, 2′-F, PS	2	Terminated [NCT02604212]
Srivast et al. (2023)	Fitusiran	Severe haemophilia a or B	Rare	79; 40	9	GalNAc	2′-OMe, 2′-F, PS	3	Approved (Qfitlia) [NCT03417245]
Young et al. (2023)	Fitusiran	Severe haemophilia a or B	Rare	41; 19	9	GalNAc	2′-OMe, 2′-F, PS	3	Approved (Qfitlia) [NCT03417102]
Hou et al. (2024)	Xalnesiran (RG6346)	Chronic HBV infection	Common	60; 35	24	GalNAc	2′-OMe, 2′-F, PS	2	Discontinued [NCT04225715]
Doi et al. (2023)	TRK-250	Idiopathic pulmonary fibrosis	Rare	24; 10	1	Unspecified	Undisclosed	1	Ongoing [NCT03727802]
Koren et al. (2024)	Inclisiran	ASCVD	Common	234; 216	11	GalNAc	2′-OMe, 2′-F, PS	3	Approved (LEQVIO) [NCT04929249]
Taub et al. (2025)	Inclisiran	Elevated LDL-C	Common	174; 87	6	GalNAc	2′-OMe, 2′-F, PS	3	Approved (LEQVIO) [NCT05763875]

*, †, ‡, § = Studies that include 2 trials in one article.

Tx (Treatment group), Co (Comparison group), mo (Month), HoFH (Homozygous Familial Hypercholesterolemia), HeFH (Heterozygous Familial Hypercholesterolemia), ASCVD (Atherosclerotic Cardiovascular Disease), AKI (Acute Kidney Injury), IgA nephropathy (Immunoglobulin A nephropathy), PH1 (Primary Hyperoxaluria Type 1), PH2 (Primary Hyperoxaluria Type 2), PH3 (Primary Hyperoxaluria Type 3), Lp(a) (Lipoprotein(a)), LDL-C (Low-Density Lipoprotein Cholesterol), FCS (Familial Chylomicronemia Syndrome), hATTR amyloidosis (hereditary transthyretin amyloidosis), ATTR-CM (Transthyretin Amyloidosis with cardiomyopathy), hATTR-CM (hereditary transthyretin amyloidosis with cardiomyopathy), HBV (Hepatitis B Virus), BOS (Bronchiolitis Obliterans Syndrome), RSV (Respiratory Syncytial Virus), LTx (Lung Transplantation), MAFLD (Metabolic dysfunction-associated steatotic liver disease), 2′-OMe (2′-O-methyl), 2′-F (2′-deoxy-2′-fluoro), PS (Phosphorothioate), 2′-O-MOE (2′-O-methoxyethyl), S-GNA ((S)-glycol nucleic acid), GalNAc (N-acetylgalactosamine).

### Quality assessment

The McHarm score ranged from 7 to 14, with a median score of 13 (Supplementary Material S3). Of the 53 reports, 51 (96%) reports specified the number of deaths in each study group, and 50 reports (94%) provided a precise definition of serious adverse events either in the main article or the protocol.

### Safety outcomes

**FIGURE 2:**
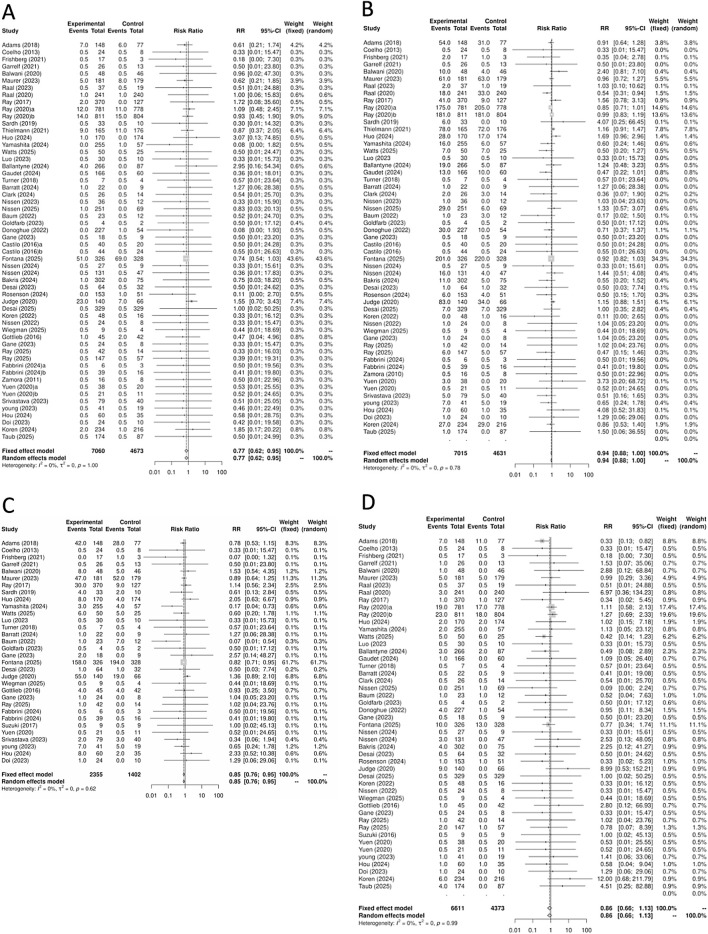
Forest plot for the safety outcomes. **(A)** Death **(B)** serious adverse event **(C)** severe adverse event **(D)** discontinuation due to adverse event.

A meta-analysis of safety outcomes was performed on the 57 RCTs. Among the 57 RCTs, one study did not report data on mortality (Suzuki et al., 2017). Consequently, the analysis included data from 56 studies for death, 55 for SAEs, 32 for severe adverse events, and 48 for discontinuation because of adverse events. Compared to the control group, the siRNA treatment group did not have an increased risk of death or severe adverse events (RR 0.77, 95% CI 0.62–0.95, *I* ^*2*^ = 0% and RR 0.85, 95% CI 0.76–0.95, *I* ^*2*^ = 0%, respectively). The risk of SAEs and treatment discontinuation due to adverse events did not show a significant increase in the treatment group compared with the control group (RR 0.94, 95% CI 0.88–1.00, *I* ^*2*^ = 0% and RR 0.86, 95% CI 0.66–1.13, *I* ^*2*^ = 0%, respectively) (Figure 2).

### Publication bias

Visual inspection of the funnel plot for the 56 studies reporting on death revealed no significant asymmetry, suggesting a low likelihood of publication bias (Supplementary Material S4).

### Subgroup analysis of death outcome

Subgroup analysis based on disease type showed that siRNA treatment did not increase the risk of death compared with the control in either the rare disease subgroup (RR 0.76, 95% CI 0.58–1.00) or the common disease subgroup (RR 0.78, 95% CI 0.54–1.12). Consistent results were observed in subgroup analyses by drug delivery technology (GalNAc vs. non-GalNAc) and clinical trial phase (Phase 1, 2, or 3), with siRNA treatment not increasing the risk of death in any subgroup. When analyzed by development status (marketed, ongoing, or discontinued/terminated), the risk of death was not increased in the marketed and ongoing development subgroups (RR 0.76, 95% CI 0.59–0.96 and RR 0.47, 95% CI 0.22–0.98, respectively). The discontinued/terminated subgroup showed a risk of death similar to that of the control group (RR 1.01, 95% CI 0.60–1.71) (Table 2).

**TABLE 2:** Subgroup analysis of death according to disease category, delivery mechanism, study stage, and development status.

Subgroups	No. Of trials (Tn/Cn)	RR (95% CI)
Disease category		
Rare disease	20 (1,265/888)	0.76 (0.58–1.00)
Common disease	36 (5,795/3,785)	0.78 (0.54–1.12)
Delivery mechanism		
GalNAc	44 (6,307/4,094)	0.79 (0.62–1.01)
Others	12 (753/579)	0.66 (0.40–1.11)
Study stage		
Phase 1	15 (426/163)	0.40 (0.15–1.06)
Phase 2	24 (3,138/1,395)	0.61 (0.35–1.06)
Phase 3	17 (3,496/3,115)	0.84 (0.66–1.06)
Product development status		
Marketed	22 (4,038/3,245)	0.76 (0.59–0.96)
Ongoing development	24 (2,488/1,049)	0.47 (0.22–0.98)
Discontinued or terminated	10 (534/379)	1.01 (0.60–1.71)

No (number), Tn (Treatment group), Cn (Comparison group), OR (Odds ratio), CI (Confidence interval), GalNAc (N-acetylgalactosamine).

## Discussion

This systematic review and meta-analysis assessed the safety of siRNA therapeutics by analyzing 57 randomized clinical trials covering 28 different agents and a total of 11,757 participants. We evaluated the safety outcomes and further investigated the safety profile through subgroup analyses based on disease type, clinical trial phase, drug development status, and the use of GalNAc conjugation. The overall quality of safety data reporting was high, with a median McHarm score of 13, and 38 reports (71.7%) scored 12 or higher. This indicates that the quality of adverse event reporting was sufficient to justify an evaluation of the safety of siRNA agents using the available data.

Our analysis revealed that the siRNA therapeutic group did not show a significant increase in the incidence of key safety outcomes (death, serious adverse events, severe adverse events, and treatment discontinuation due to adverse events) compared with the control group. In fact, the incidence of death and severe adverse events was significantly lower in the treatment group. Nevertheless, this finding from the analysis of the overall group does not preclude the possibility of potential toxicity in individual patients. Some clinical trials have reported serious hepatic or electrocardiogram abnormalities that led to participant withdrawal (Balwani et al., 2020; Baum et al., 2023). Although rare, these cases underscore the necessity of rigorous monitoring for adverse events where a causal link to the drug cannot be excluded.

The risk-benefit assessment for siRNA therapeutics in common diseases may differ from that for rare, often genetic, diseases (Goyenvalle et al., 2023; Andersson, 2022). This difference is partly because multiple therapeutic alternatives often exist for common conditions (Goyenvalle et al., 2023). Therefore, the safety threshold is particularly high. Even with superior efficacy, siRNA therapeutics may not achieve widespread clinical adoption without a well-established safety profile. Our subgroup analysis for common diseases showed that siRNA treatment did not increase mortality risk (RR = 0.78), suggesting that its use in this population is unlikely to be associated with high risk. However, this finding has limitations, as most of the included studies enrolled patients who had failed standard therapies and thus had higher disease severity. Therefore, an extrapolation to patient populations with lower disease severity should be done with caution. To address this, trials like the VICTORION-MONO study are evaluating the strategy of administering inclisiran earlier before other lipid-lowering therapies are used (Taub et al., 2025). Such study designs will enable a more accurate risk-benefit assessment of siRNA therapeutics in a broader population with common diseases.

From the perspective of delivery mechanisms, GalNAc conjugation, which is widely applied to both marketed and investigational drugs, demonstrated an acceptable safety profile in terms of mortality. Notably, therapeutics using non-GalNAc delivery technologies also did not show an increase in mortality (RR: 0.66, 95% CI: 0.40–1.11). However, this latter finding must be interpreted with caution. It is possible that non-GalNAc drugs with unfavorable safety profiles were discontinued in early clinical or preclinical phases and the data were never published. This potential for publication bias limits the ability to conclude that non-GalNAc delivery methods have a similarly acceptable safety profile as GalNAc delivery methods. When stratified by development status, marketed siRNA drugs demonstrated no increased mortality compared with placebo, reaffirming their clinical utility. Agents currently in development also showed a favorable mortality profile without increased risk (RR 0.47), indicating a promising safety profile. Given that most of the data for these investigational agents comes from early-phase trials (9 Phase 1 and 14 Phase 2 studies), with only one Phase 3 study (Watts et al., 2025), a more precise safety profile will be established as data from large-scale Phase 3 trials accumulate.

The FDA’s draft guidance for the preclinical development of siRNA agents outlines the required *in vitro* and *in vivo* assessments, such as on/off-target toxicities, PK/PD, general toxicity, and carcinogenicity (USFaD, 2024). The toxicities considered during this phase can be broadly classified into three types: (Chaud et al., 2025): hybridization- and sequence-dependent, (Levin, 2017), hybridization-independent but sequence-dependent, and (Ranjbar et al., 2023) hybridization- and sequence-independent (Goyenvalle et al., 2023; Andersson, 2022). Hybridization- and sequence-dependent toxicities, such as off-target effects, are managed preclinically via *in silico* analysis (Lindow et al., 2012; Rider et al., 2022). Hybridization- and sequence-independent toxicities, which occur when plasma concentrations exceed a certain threshold, can be managed by adjusting dosage and frequency (Goyenvalle et al., 2023). The remaining category—hybridization-independent but sequence-dependent effects includes proinflammatory effects and toxicity to high-exposure organs like the liver and kidneys (Goyenvalle et al., 2023). Proinflammatory effects, such as injection site reactions (ISRs), can still occur despite chemical modifications (Chi et al., 2017; Mustonen et al., 2017). ISRs were the most frequently reported adverse event in the included RCTs. However, the vast majority were of mild-to-moderate severity and resolved without intervention. Similarly, toxicity to high-exposure organs, assessed via laboratory results (e.g., AST/ALT, creatinine), was largely characterized by changes that were not clinically relevant or were transient, mild elevations that resolved spontaneously. The acceptable safety profile observed in the RCT outcomes, combined with the finding that most ISRs and lab abnormalities were predictable and manageable, implies that the adverse events reported in clinical trials generally fell within the anticipated range of toxicities considered during preclinical safety assessments. It is important, though, to interpret this with caution. Because our meta-analysis relies exclusively on published clinical data, preclinical or early toxicity profiles remain unreported. Therefore, it should be conservatively interpreted as reflecting the improved clinical tolerability of newer siRNA platforms.

Despite preclinical frameworks to anticipate risks, incompletely understood toxicity mechanisms could lead to unforeseen safety issues in the clinical setting (Ranjbar et al., 2023). This is particularly challenging in intractable diseases, where determining the specific cause of death and its association with an intervention can be difficult. Among several potential causes, imperfections in the chemical composition of agents have been identified, and efforts to address this are ongoing (Ali Zaidi et al., 2023; Zhang et al., 2021). Of the four studies in our analysis that were discontinued because of safety concerns, three were halted because the delivery vehicle, rather than the siRNA molecule itself, was found to cause mortality in non-human primates (Turner et al., 2018; Yuen et al., 2020). The fourth study, involving revusiran, was terminated because of a mortality imbalance (23 deaths in the treatment group vs. 7 in the placebo group) by the end of the safety follow-up period (Judge et al., 2020). While a definitive cause for the imbalance could not be identified, the presence of imbalances in other adverse events suggested that drug-mediated toxicity was likely a contributing factor (Judge et al., 2020). Revusiran is a first-generation GalNAc-siRNA conjugate with “standard template chemistry,” where 2′-OMe and 2′-F modifications alternate on every ribose (Judge et al., 2020). Vutrisiran, which targets the same indication, is a second-generation compound with “enhanced stabilized chemistry” (ESC) that modulates the amount of ribose modification, leading to enhanced resistance to degradation (Foster et al., 2018). This difference in structural stability results in markedly different drug exposures: the administration of revusiran involves 500-mg subcutaneous injection weekly, whereas vutrisiran is administered as a 25-mg subcutaneous injection once every 12 weeks (Fontana et al., 2025; Judge et al., 2020). All other approved and most ongoing siRNA agents now use second-generation ESC modifications. The favorable safety results of these other ongoing therapies suggest that improvements in delivery systems and chemical modifications, such as GalNAc conjugation and ESC, have translated into enhanced clinical safety.

This study has several limitations. First, by including only RCTs, our analysis may lack studies with very large sample sizes or long follow-up periods. Among the included RCTs, only three had a follow-up duration of 2 years or more (Huo et al., 2024; Clark et al., 2024; Fontana et al., 2025). For rare diseases with small recruitment pools, the statistical power to properly evaluate uncommon outcomes like death may be limited. Phase 1 and 2 trials involve smaller cohorts and shorter follow-ups, which may dilute the overall pooled estimates or limit the detection of rare adverse events. Nevertheless, we exclusively included RCTs because they are the gold standard for statistically verifying differences in safety outcomes between intervention and control groups (Chou et al., 2010). Given the ethical challenges of maintaining a long-term placebo-controlled phase for patients with high unmet medical needs, tracking safety outcomes in open-label extension (OLE) phases is needed for assessing long-term safety. A 5-year OLE study of inclisiran found no new safety signals and reported that most instances of elevated liver enzymes, a potential issue with repeated dosing, were of mild or moderate severity (Ray et al., 2023). Second, as safety was often a secondary endpoint in many RCTs, safety may not have been reported with the same level of detail as the primary efficacy endpoint. In fact, severe adverse events were not explicitly reported in 24 of the 53 reports. However, several factors support the reliability of the safety data. Most RCTs specified safety assessment plans in their protocols and provided precise definitions for adverse events. For death and SAEs, protocols mandated immediate reporting (e.g., within 24 h), minimizing the chance of missed reports. Furthermore, the use of standardized dictionaries like Medical Dictionary for Regulatory Activities and Common Terminology Criteria for Adverse Events for collecting and classifying adverse events ensured data consistency. We therefore conclude that the reporting of safety outcomes was systematic and reliable. Lastly, while we attempted to address the heterogeneity of diverse trials through subgroup analyses based on disease type, drug development status, and the use of GalNAc conjugation, our study could not evaluate all pharmacological variables. Due to limited number of trials and low adverse event rates across highly specific subgroups, we were unable to conduct further stratified analyses by dosing schedules, or distinct non-GalNAc delivery systems such as lipid nanoparticles (LNPs). Future meta-analyses with larger pools of standardized data are needed to evaluate how these pharmacological factors influence the safety profile of siRNA agents.

Despite these limitations, this study holds significant value as the first systematic review and meta-analysis to quantitatively evaluate key safety outcomes of siRNA therapeutics across a diverse range of diseases. A further strength is the use of subgroup analyses based on specific criteria, such as disease rarity and development status, to compare safety under different conditions. Finally, this research offers a unique contribution by using clinical trial safety outcomes to reflect on the adequacy of the preclinical safety framework for siRNA therapeutics.

## Conclusion

This systematic review and meta-analysis confirmed that siRNA therapeutics do not significantly increase the risk of safety outcomes compared with controls. This finding supports that the advancements of chemical modifications and drug delivery systems, introduced to reduce the toxicity of early-generation siRNAs, has successfully translated into enhanced clinical safety. Therefore, these results underscore the critical importance of maintaining and refining these toxicity mitigation strategies in the development of next-generation siRNA therapeutics. Nevertheless, as unforeseen safety issues can still manifest during clinical development, thorough safety validation throughout the early clinical trial phases remains essential. While short-term safety profile is reassuring, extended follow-up surveillance of delayed toxicities remain necessary to elucidate the long-term safety profile of these agents.

## Data Availability

The data analyzed in this study is subject to the following licenses/restrictions: If a researcher requests data, it can be obtained through the corresponding authors. Requests to access these datasets should be directed to Hyunsuk Jeong, suejeong@catholic.ac.kr.

## References

[B1] Adams D. Gonzalez-Duarte A. O'Riordan W. D. Yang C. C. Ueda M. Kristen A. V. (2018). Patisiran, an RNAi therapeutic, for hereditary transthyretin amyloidosis. N. Engl. J. Med. 379, 11–21. 10.1056/NEJMoa1716153 29972753

[B2] Ali Zaidi S. S. Fatima F. Ali Zaidi S. A. Zhou D. Deng W. Liu S. (2023). Engineering siRNA therapeutics: challenges and strategies. J. Nanobiotechnology 21, 381. 10.1186/s12951-023-02147-z 37848888 PMC10583313

[B3] Alshaer W. Zureigat H. Al Karaki A. Al-Kadash A. Gharaibeh L. Hatmal M. M. (2021). siRNA: mechanism of action, challenges, and therapeutic approaches. Eur. J. Pharmacol. 905, 174178. 10.1016/j.ejphar.2021.174178 34044011

[B4] Anand P. Zhang Y. Patil S. Kaur K. (2025). Metabolic stability and targeted delivery of oligonucleotides: advancing RNA therapeutics beyond the liver. J. Med. Chem. 68, 6870–6896. 10.1021/acs.jmedchem.4c02528 39772535 PMC11998008

[B5] Andersson P. (2022). Preclinical safety assessment of therapeutic oligonucleotides. Editors V. Arechavala-Gomeza. New York, NY: Humana Press. 10.1007/978-1-0716-2010-6_25 PMC9703267 35213031

[B6] Bakris G. L. Saxena M. Gupta A. Chalhoub F. Lee J. Stiglitz D. (2024). RNA interference with zilebesiran for mild to moderate hypertension: the KARDIA-1 randomized clinical trial. JAMA 331, 740–749. 10.1001/jama.2024.0728 38363577 PMC10873804

[B7] Ballantyne C. M. Vasas S. Azizad M. Clifton P. Rosenson R. S. Chang T. (2024). Plozasiran, an RNA interference agent targeting APOC3, for mixed hyperlipidemia. N. Engl. J. Med. 391, 899–912. 10.1056/NEJMoa2404143 38804517

[B8] Balwani M. Sardh E. Ventura P. Peiro P. A. Rees D. C. Stolzel U. (2020). Phase 3 trial of RNAi therapeutic givosiran for acute intermittent porphyria. N. Engl. J. Med. 382, 2289–2301. 10.1056/NEJMoa1913147 32521132

[B9] Barratt J. Liew A. Yeo S. C. Fernstrom A. Barbour S. J. Sperati C. J. (2024). Phase 2 trial of cemdisiran in adult patients with IgA nephropathy: a randomized controlled trial. Clin. J. Am. Soc. Nephrol. 19, 452–462. 10.2215/CJN.0000000000000384 38214599 PMC11020434

[B10] Baum M. A. Langman C. Cochat P. Lieske J. C. Moochhala S. H. Hamamoto S. (2023). PHYOX2: a pivotal randomized study of nedosiran in primary hyperoxaluria type 1 or 2. Kidney Int. 103, 207–217. 10.1016/j.kint.2022.07.025 36007597

[B11] Benitez-Del-Castillo J. M. Moreno-Montanes J. Jimenez-Alfaro I. Munoz-Negrete F. J. Turman K. Palumaa K. (2016). Safety and efficacy clinical trials for SYL1001, a novel short interfering RNA for the treatment of dry eye disease. Invest Ophthalmol. Vis. Sci. 57, 6447–6454. 10.1167/iovs.16-20303 27893109

[B12] Chaudhary A. Kumar V. (2025). Rare diseases: a comprehensive literature review and future directions. J. Rare Dis. 4, 4. 10.1007/s44162-025-00099-6

[B13] Chi X. Gatti P. Papoian T. (2017). Safety of antisense oligonucleotide and siRNA-based therapeutics. Drug Discov. Today 22, 823–833. 10.1016/j.drudis.2017.01.013 28159625

[B14] Chou R. Aronson N. Atkins D. Ismaila A. S. Santaguida P. Smith D. H. (2010). AHRQ series paper 4: assessing harms when comparing medical interventions: AHRQ and the effective health-care program. J. Clin. Epidemiol. 63, 502–512. 10.1016/j.jclinepi.2008.06.007 18823754

[B15] Clark V. C. Strange C. Strnad P. Sanchez A. J. Kwo P. Pereira V. M. (2024). Fazirsiran for adults with Alpha-1 antitrypsin deficiency liver disease: a phase 2 placebo controlled trial (SEQUOIA). Gastroenterology 167, 1008–18 e5. 10.1053/j.gastro.2024.06.028 38964420

[B16] Coelho T. Adams D. Silva A. Lozeron P. Hawkins P. N. Mant T. (2013). Safety and efficacy of RNAi therapy for transthyretin amyloidosis. N. Engl. J. Med. 369, 819–829. 10.1056/NEJMoa1208760 23984729

[B17] Desai A. S. Webb D. J. Taubel J. Casey S. Cheng Y. Robbie G. J. (2023). Zilebesiran, an RNA interference therapeutic agent for hypertension. N. Engl. J. Med. 389, 228–238. 10.1056/NEJMoa2208391 37467498

[B18] Desai A. S. Karns A. D. Badariene J. Aswad A. Neutel J. M. Kazi F. (2025). Add-On treatment with zilebesiran for inadequately controlled hypertension: the KARDIA-2 randomized clinical trial. JAMA 334, 46–55. 10.1001/jama.2025.6681 40434761 PMC12120674

[B19] Doi H. Atsumi J. Baratz D. Miyamoto Y. (2023). A phase I study of TRK-250, a novel siRNA-Based oligonucleotide, in patients with idiopathic pulmonary fibrosis. J. Aerosol Med. Pulm. Drug Deliv. 36, 300–308. 10.1089/jamp.2023.0014 37738329

[B20] Fabbrini E. Rady B. Koshkina A. Jeon J. Y. Ayyar V. S. Gargano C. (2024). Phase 1 trials of PNPLA3 siRNA in I148M homozygous patients with MAFLD. N. Engl. J. Med. 391, 475–476. 10.1056/NEJMc2402341 39083780

[B21] Fontana M. Berk J. L. Gillmore J. D. Witteles R. M. Grogan M. Drachman B. (2025). Vutrisiran in patients with transthyretin amyloidosis with cardiomyopathy. N. Engl. J. Med. 392, 33–44. 10.1056/NEJMoa2409134 39213194

[B22] Foster D. J. Brown C. R. Shaikh S. Trapp C. Schlegel M. K. Qian K. (2018). Advanced siRNA designs further improve *in* Vivo performance of GalNAc-siRNA conjugates. Mol. Ther. 26, 708–717. 10.1016/j.ymthe.2017.12.021 29456020 PMC5910670

[B23] Frishberg Y. Deschenes G. Groothoff J. W. Hulton S. A. Magen D. Harambat J. (2021). Phase 1/2 study of lumasiran for treatment of primary hyperoxaluria type 1: a placebo-controlled randomized clinical trial. Clin. J. Am. Soc. Nephrol. 16, 1025–1036. 10.2215/CJN.14730920 33985991 PMC8425611

[B24] Gane E. J. Kim W. Lim T. H. Tangkijvanich P. Yoon J. H. Sievert W. (2023). First-in-human randomized study of RNAi therapeutic RG6346 for chronic hepatitis B virus infection. J. Hepatol. 79, 1139–1149. 10.1016/j.jhep.2023.07.026 37524230

[B25] Gane E. Lim Y. S. Kim J. B. Jadhav V. Shen L. Bakardjiev A. I. (2023). Evaluation of RNAi therapeutics VIR-2218 and ALN-HBV for chronic hepatitis B: results from randomized clinical trials. J. Hepatol. 79, 924–932. 10.1016/j.jhep.2023.05.023 37290591

[B26] Garrelfs S. F. Frishberg Y. Hulton S. A. Koren M. J. O'Riordan W. D. Cochat P. (2021). Lumasiran, an RNAi therapeutic for primary hyperoxaluria type 1. N. Engl. J. Med. 384, 1216–1226. 10.1056/NEJMoa2021712 33789010

[B27] Gaudet D. Pall D. Watts G. F. Nicholls S. J. Rosenson R. S. Modesto K. (2024). Plozasiran (ARO-APOC3) for severe hypertriglyceridemia: the SHASTA-2 randomized clinical trial. JAMA Cardiol. 9, 620–630. 10.1001/jamacardio.2024.0959 38583092 PMC11000138

[B28] Goldfarb D. S. Lieske J. C. Groothoff J. Schalk G. Russell K. Yu S. (2023). Nedosiran in primary hyperoxaluria subtype 3: results from a phase I, single-dose study (PHYOX4). Urolithiasis 51, 80. 10.1007/s00240-023-01453-3 37118061 PMC10147791

[B29] Gottlieb J. Zamora M. R. Hodges T. Musk A. W. Sommerwerk U. Dilling D. (2016). ALN-RSV01 for prevention of bronchiolitis Obliterans syndrome after respiratory syncytial virus infection in lung transplant recipients. J. Heart Lung Transpl. 35, 213–221. 10.1016/j.healun.2015.08.012 26452996

[B30] Goyenvalle A. Jimenez-Mallebrera C. van Roon W. Sewing S. Krieg A. M. Arechavala-Gomeza V. (2023). Considerations in the preclinical assessment of the safety of antisense oligonucleotides. Nucleic Acid. Ther. 33, 1–16. 10.1089/nat.2022.0061 36579950 PMC9940817

[B31] Hou J. Zhang W. Xie Q. Hua R. Tang H. Morano A. L. E. (2024). Xalnesiran with or without an immunomodulator in chronic hepatitis B. N. Engl. J. Med. 391, 2098–2109. 10.1056/NEJMoa2405485 39774313

[B32] Huo Y. Lesogor A. Lee C. W. Chiang C. E. Mena-Madrazo J. Poh K. K. (2024). Efficacy and safety of inclisiran in Asian patients: results from ORION-18. JACC Asia 4 (4), 123–134. 10.1016/j.jacasi.2023.09.006 38371290 PMC10866732

[B33] Janas M. M. Harbison C. E. Perry V. K. Carito B. Sutherland J. E. Vaishnaw A. K. (2018). The nonclinical safety profile of GalNAc-conjugated RNAi therapeutics in subacute studies. Toxicol. Pathol. 46, 735–745. 10.1177/0192623318792537 30139307 PMC6249674

[B34] Jeon J. Y. Ayyar V. S. Mitra A. (2022). Pharmacokinetic and pharmacodynamic modeling of siRNA therapeutics - a minireview. Pharm. Res. 39, 1749–1759. 10.1007/s11095-022-03333-8 35819583

[B35] Judge A. D. Bola G. Lee A. C. MacLachlan I. (2006). Design of noninflammatory synthetic siRNA mediating potent gene silencing *in* vivo. Mol. Ther. 13, 494–505. 10.1016/j.ymthe.2005.11.002 16343994

[B36] Judge D. P. Kristen A. V. Grogan M. Maurer M. S. Falk R. H. Hanna M. (2020). Phase 3 multicenter study of revusiran in patients with hereditary transthyretin-mediated (hATTR) amyloidosis with cardiomyopathy (ENDEAVOUR). Cardiovasc Drugs Ther. 34, 357–370. 10.1007/s10557-019-06919-4 32062791 PMC7242280

[B37] Khvorova A. Watts J. K. (2017). The chemical evolution of oligonucleotide therapies of clinical utility. Nat. Biotechnol. 35, 238–248. 10.1038/nbt.3765 28244990 PMC5517098

[B38] Koren M. J. Moriarty P. M. Baum S. J. Neutel J. Hernandez-Illas M. Weintraub H. S. (2022). Preclinical development and phase 1 trial of a novel siRNA targeting lipoprotein(a). Nat. Med. 28, 96–103. 10.1038/s41591-021-01634-w 35027752

[B39] Koren M. J. Rodriguez F. East C. Toth P. P. Watwe V. Abbas C. A. (2024). An “Inclisiran First” strategy vs usual care in patients with atherosclerotic cardiovascular disease. J. Am. Coll. Cardiol. 83, 1939–1952. 10.1016/j.jacc.2024.03.382 38593947

[B40] Levin A. A. (2017). Targeting therapeutic oligonucleotides. N. Engl. J. Med. 376, 86–88. 10.1056/NEJMcibr1613559 28052219

[B41] Lindow M. Vornlocher H. P. Riley D. Kornbrust D. J. Burchard J. Whiteley L. O. (2012). Assessing unintended hybridization-induced biological effects of oligonucleotides. Nat. Biotechnol. 30, 920–923. 10.1038/nbt.2376 23051805

[B42] Luo Z. Huang Z. Sun F. Guo F. Wang Y. Kao S. (2023). The clinical effects of inclisiran, a first-in-class LDL-C lowering siRNA therapy, on the LDL-C levels in Chinese patients with hypercholesterolemia. J. Clin. Lipidol. 17, 392–400. 10.1016/j.jacl.2023.04.010 37164838

[B43] Maurer M. S. Kale P. Fontana M. Berk J. L. Grogan M. Gustafsson F. (2023). Patisiran treatment in patients with transthyretin cardiac amyloidosis. N. Engl. J. Med. 389, 1553–1565. 10.1056/NEJMoa2300757 37888916 PMC10757426

[B44] Mustonen E. K. Palomaki T. Pasanen M. (2017). Oligonucleotide-based pharmaceuticals: non-Clinical and clinical safety signals and non-clinical testing strategies. Regul. Toxicol. Pharmacol. 90, 328–341. 10.1016/j.yrtph.2017.09.028 28966105

[B45] Nair J. K. Willoughby J. L. Chan A. Charisse K. Alam M. R. Wang Q. (2014). Multivalent N-acetylgalactosamine-conjugated siRNA localizes in hepatocytes and elicits robust RNAi-mediated gene silencing. J. Am. Chem. Soc. 136, 16958–16961. 10.1021/ja505986a 25434769

[B46] Nissen S. E. Wolski K. Balog C. Swerdlow D. I. Scrimgeour A. C. Rambaran C. (2022). Single ascending dose study of a short interfering RNA targeting lipoprotein(a) production in individuals with elevated plasma lipoprotein(a) levels. JAMA 327, 1679–1687. 10.1001/jama.2022.5050 35368052 PMC8978050

[B47] Nissen S. E. Linnebjerg H. Shen X. Wolski K. Ma X. Lim S. (2023). Lepodisiran, an extended-duration short interfering RNA targeting lipoprotein(a): a randomized dose-ascending clinical trial. JAMA 330, 2075–2083. 10.1001/jama.2023.21835 37952254 PMC10641766

[B48] Nissen S. E. Wolski K. Watts G. F. Koren M. J. Fok H. Nicholls S. J. (2024a). Single ascending and multiple-dose trial of zerlasiran, a short interfering RNA targeting lipoprotein(a): a randomized clinical trial. JAMA 331, 1534–1543. 10.1001/jama.2024.4504 38587822 PMC11002768

[B49] Nissen S. E. Wang Q. Nicholls S. J. Navar A. M. Ray K. K. Schwartz G. G. (2024b). Zerlasiran-A small-interfering RNA targeting lipoprotein(a): a phase 2 randomized clinical trial. JAMA 332, 1992–2002. 10.1001/jama.2024.21957 39556769 PMC11574722

[B50] Nissen S. E. Ni W. Shen X. Wang Q. Navar A. M. Nicholls S. J. (2025). Lepodisiran - a long-duration small interfering RNA targeting lipoprotein(a). N. Engl. J. Med. 392, 1673–1683. 10.1056/NEJMoa2415818 40162643

[B51] O'Donoghue M. L. Rosenson R. S. Gencer B. Lopez J. A. G. Lepor N. E. Baum S. J. (2022). Small interfering RNA to reduce lipoprotein(a) in cardiovascular disease. N. Engl. J. Med. 387, 1855–1864. 10.1056/NEJMoa2211023 36342163

[B52] Patzel V. (2007). *In silico* selection of active siRNA. Drug Discov. Today 12, 139–148. 10.1016/j.drudis.2006.11.015 17275734

[B53] Raal F. J. Kallend D. Ray K. K. Turner T. Koenig W. Wright R. S. (2020). Inclisiran for the treatment of heterozygous familial hypercholesterolemia. N. Engl. J. Med. 382, 1520–1530. 10.1056/NEJMoa1913805 32197277

[B54] Raal F. Durst R. Bi R. Talloczy Z. Maheux P. Lesogor A. (2023). Efficacy, safety, and tolerability of inclisiran in patients with homozygous familial hypercholesterolemia: results from the ORION-5 randomized clinical trial. Circulation 149, 354–362. 10.1161/CIRCULATIONAHA.122.063460 37850379 PMC10815002

[B55] Ranjbar S. Zhong X. B. Manautou J. Lu X. (2023). A holistic analysis of the intrinsic and delivery-mediated toxicity of siRNA therapeutics. Adv. Drug Deliv. Rev. 201, 115052. 10.1016/j.addr.2023.115052 37567502 PMC10543595

[B56] Ray K. K. Landmesser U. Leiter L. A. Kallend D. Dufour R. Karakas M. (2017). Inclisiran in patients at high cardiovascular risk with elevated LDL cholesterol. N. Engl. J. Med. 376, 1430–1440. 10.1056/NEJMoa1615758 28306389

[B57] Ray K. K. Wright R. S. Kallend D. Koenig W. Leiter L. A. Raal F. J. (2020). Two phase 3 trials of inclisiran in patients with elevated LDL cholesterol. N. Engl. J. Med. 382, 1507–1519. 10.1056/NEJMoa1912387 32187462

[B58] Ray K. K. Troquay R. P. T. Visseren F. L. J. Leiter L. A. Scott Wright R. Vikarunnessa S. (2023). Long-term efficacy and safety of inclisiran in patients with high cardiovascular risk and elevated LDL cholesterol (ORION-3): results from the 4-year open-label extension of the ORION-1 trial. Lancet Diabetes Endocrinol. 11, 109–119. 10.1016/S2213-8587(22)00353-9 36620965

[B59] Ray K. K. Linnebjerg H. Michael L. F. Shen X. Ma X. Lim S. (2025a). Effect of ANGPTL3 inhibition with solbinsiran in preclinical and early human studies. J. Am. Coll. Cardiol. 85, 1803–1818. 10.1016/j.jacc.2025.03.005 40158211

[B60] Ray K. K. Oru E. Rosenson R. S. Jones J. Ma X. Walgren J. (2025b). Durability and efficacy of solbinsiran, a GalNAc-conjugated siRNA targeting ANGPTL3, in adults with mixed dyslipidaemia (PROLONG-ANG3): a double-blind, randomised, placebo-controlled, phase 2 trial. Lancet 405, 1594–1607. 10.1016/S0140-6736(25)00507-0 40179932

[B61] Rider D. Chivers S. Aretz J. Eisermann M. Loffler K. Hauptmann J. (2022). Preclinical toxicological assessment of A novel siRNA, SLN360, targeting elevated lipoprotein (a) in cardiovascular disease. Toxicol. Sci. 189, 237–249. 10.1093/toxsci/kfac067 35737426 PMC9516055

[B62] Roberts T. C. Langer R. Wood M. J. A. (2020). Advances in oligonucleotide drug delivery. Nat. Rev. Drug Discov. 19, 673–694. 10.1038/s41573-020-0075-7 32782413 PMC7419031

[B63] Rosenson R. S. Gaudet D. Hegele R. A. Ballantyne C. M. Nicholls S. J. Lucas K. J. (2024). Zodasiran, an RNAi therapeutic targeting ANGPTL3, for mixed hyperlipidemia. N. Engl. J. Med. 391, 913–925. 10.1056/NEJMoa2404147 38809174

[B64] Sardh E. Harper P. Balwani M. Stein P. Rees D. Bissell D. M. (2019). Phase 1 trial of an RNA interference therapy for acute intermittent porphyria. N. Engl. J. Med. 380, 549–558. 10.1056/NEJMoa1807838 30726693

[B65] Springer A. D. Dowdy S. F. (2018). GalNAc-siRNA conjugates: leading the way for delivery of RNAi therapeutics. Nucleic Acid. Ther. 28, 109–118. 10.1089/nat.2018.0736 29792572 PMC5994659

[B66] Srivastava A. Rangarajan S. Kavakli K. Klamroth R. Kenet G. Khoo L. (2023). Fitusiran prophylaxis in people with severe haemophilia A or haemophilia B without inhibitors (ATLAS-A/B): a multicentre, open-label, randomised, phase 3 trial. Lancet Haematol. 10, e322–e332. 10.1016/S2352-3026(23)00037-6 37003278

[B67] Suzuki K. Yokoyama J. Kawauchi Y. Honda Y. Sato H. Aoyagi Y. (2017). Phase 1 clinical study of siRNA targeting carbohydrate sulphotransferase 15 in crohn's disease patients with active mucosal lesions. J. Crohns Colitis 11, 221–228. 10.1093/ecco-jcc/jjw143 27484097

[B68] Taub P. R. Gutierrez A. Wewers D. Garcia Cantu E. Cao H. Deck C. (2025). Safety and lipid-lowering efficacy of inclisiran monotherapy in patients without ASCVD: the VICTORION-mono randomized clinical trial. J. Am. Coll. Cardiol. 86, 196–208. 10.1016/j.jacc.2025.04.049 40392667

[B69] Thielmann M. Corteville D. Szabo G. Swaminathan M. Lamy A. Lehner L. J. (2021). Teprasiran, a small interfering RNA, for the prevention of acute kidney injury in high-risk patients undergoing cardiac surgery: a randomized clinical study. Circulation 144, 1133–1144. 10.1161/CIRCULATIONAHA.120.053029 34474590 PMC8487715

[B70] Turner A. M. Stolk J. Bals R. Lickliter J. D. Hamilton J. Christianson D. R. (2018). Hepatic-targeted RNA interference provides robust and persistent knockdown of alpha-1 antitrypsin levels in ZZ patients. J. Hepatol. 69, 378–384. 10.1016/j.jhep.2018.03.012 29572094

[B71] UsfaD A. (2024). Nonclinical Safety Assessment of Oligonucleotide-based Therapeutics: Guidance for Industry. Silver Spring, MD: U.S. Food and Drug Administration.

[B72] Wang F. Zuroske T. Watts J. K. (2020). RNA therapeutics on the rise. Nat. Rev. Drug Discov. 19, 441–442. 10.1038/d41573-020-00078-0 32341501

[B73] Watts G. F. Rosenson R. S. Hegele R. A. Goldberg I. J. Gallo A. Mertens A. (2025). Plozasiran for managing persistent chylomicronemia and pancreatitis risk. N. Engl. J. Med. 392, 127–137. 10.1056/NEJMoa2409368 39225259

[B74] Wiegman A. Peterson A. L. Hegele R. A. Bruckert E. Schweizer A. Lesogor A. (2025). Efficacy and safety of inclisiran in adolescents with genetically confirmed homozygous familial hypercholesterolemia: results from the double-blind, placebo-controlled part of the ORION-13 randomized trial. Circulation 151, 1758–1766. 10.1161/CIRCULATIONAHA.124.073233 40391436 PMC12180692

[B75] Yamashita S. Kiyosue A. Maheux P. Mena-Madrazo J. Lesogor A. Shao Q. (2024). Efficacy, safety, and pharmacokinetics of inclisiran in Japanese patients: results from ORION-15. J. Atheroscler. Thromb. 31, 876–903. 10.5551/jat.64454 38220186 PMC11150722

[B76] Young G. Srivastava A. Kavakli K. Ross C. Sathar J. You C. W. (2023). Efficacy and safety of fitusiran prophylaxis in people with haemophilia A or haemophilia B with inhibitors (ATLAS-INH): a multicentre, open-label, randomised phase 3 trial. Lancet 401, 1427–1437. 10.1016/S0140-6736(23)00284-2 37003287

[B77] Yuen M. F. Schiefke I. Yoon J. H. Ahn S. H. Heo J. Kim J. H. (2020). RNA interference therapy with ARC-520 results in prolonged hepatitis B surface antigen response in patients with chronic hepatitis B infection. Hepatology 72, 19–31. 10.1002/hep.31008 31654573 PMC7496196

[B78] Zamora M. R. Budev M. Rolfe M. Gottlieb J. Humar A. Devincenzo J. (2011). RNA interference therapy in lung transplant patients infected with respiratory syncytial virus. Am. J. Respir. Crit. Care Med. 183, 531–538. 10.1164/rccm.201003-0422OC 20851929

[B79] Zhang M. M. Bahal R. Rasmussen T. P. Manautou J. E. Zhong X. B. (2021). The growth of siRNA-based therapeutics: updated clinical studies. Biochem. Pharmacol. 189, 114432. 10.1016/j.bcp.2021.114432 33513339 PMC8187268

