# Supplementary material for: Safety profile of small interfering RNA therapeutics: a systematic review and meta-analysis of randomized controlled trials
Source: Front Drug Saf Regul. 2026 Jul 10;6:1855572. doi: 10.3389/fdsfr.2026.1855572 (PMC13396009; doi:10.3389/fdsfr.2026.1855572)
Supplement: Supplementary file 1 [file DataSheet1.pdf]

Supplementary 1. PRISM checklists

Section and Topic	Item #	Checklist item	Location where item is reported
**TITLE**			
Title	1	Identify the report as a systematic review.	Title
**ABSTRACT**			
Abstract	2	See the PRISMA 2020 for Abstracts checklist.	Abstract
**INTRODUCTION**			
Rationale	3	Describe the rationale for the review in the context of existing knowledge.	Introduction
Objectives	4	Provide an explicit statement of the objective(s) or question(s) the review addresses.	Introduction
**METHODS**			
Eligibility criteria	5	Specify the inclusion and exclusion criteria for the review and how studies were grouped for the syntheses.	Method
Information sources	6	Specify all databases, registers, websites, organisations, reference lists and other sources searched or consulted to identify studies. Specify the date when each source was last searched or consulted.	Method
Search strategy	7	Present the full search strategies for all databases, registers and websites, including any filters and limits used.	Method
Selection process	8	Specify the methods used to decide whether a study met the inclusion criteria of the review, including how many reviewers screened each record and each report retrieved, whether they worked independently, and if applicable, details of automation tools used in the process.	Method
Data collection process	9	Specify the methods used to collect data from reports, including how many reviewers collected data from each report, whether they worked independently, any processes for obtaining or confirming data from study investigators, and if applicable, details of automation tools used in the process.	Method
Data items	10a	List and define all outcomes for which data were sought. Specify whether all results that were compatible with each outcome domain in each study were sought (e.g. for all measures, time points, analyses), and if not, the methods used to decide which results to collect.	Method
	10b	List and define all other variables for which data were sought (e.g. participant and intervention characteristics, funding sources). Describe any assumptions made about any missing or unclear information.	Method
Study risk of bias assessment	11	Specify the methods used to assess risk of bias in the included studies, including details of the tool(s) used, how many reviewers assessed each study and whether they worked independently, and if applicable, details of automation tools used in the process.	Method
Effect measures	12	Specify for each outcome the effect measure(s) (e.g. risk ratio, mean difference) used in the synthesis or presentation of results.	Method
Synthesis methods	13a	Describe the processes used to decide which studies were eligible for each synthesis (e.g. tabulating the study intervention characteristics and comparing against the planned groups for each synthesis (item #5)).	Method
	13b	Describe any methods required to prepare the data for presentation or synthesis, such as handling of missing summary statistics, or data conversions.	NA
	13c	Describe any methods used to tabulate or visually display results of individual studies and syntheses.	NA
	13d	Describe any methods used to synthesize results and provide a	Results

Section and Topic	Item #	Checklist item	Location where item is reported
		rationale for the choice(s). If meta-analysis was performed, describe the model(s), method(s) to identify the presence and extent of statistical heterogeneity, and software package(s) used.	
	13e	Describe any methods used to explore possible causes of heterogeneity among study results (e.g. subgroup analysis, meta-regression).	Results
	13f	Describe any sensitivity analyses conducted to assess robustness of the synthesized results.	NA
Reporting bias assessment	14	Describe any methods used to assess risk of bias due to missing results in a synthesis (arising from reporting biases).	NA
Certainty assessment	15	Describe any methods used to assess certainty (or confidence) in the body of evidence for an outcome.	NA
**RESULTS**			
Study selection	16a	Describe the results of the search and selection process, from the number of records identified in the search to the number of studies included in the review, ideally using a flow diagram.	Results
	16b	Cite studies that might appear to meet the inclusion criteria, but which were excluded, and explain why they were excluded.	Results
Study characteristics	17	Cite each included study and present its characteristics.	Results
Risk of bias in studies	18	Present assessments of risk of bias for each included study.	Results
Results of individual studies	19	For all outcomes, present, for each study: (a) summary statistics for each group (where appropriate) and (b) an effect estimate and its precision (e.g. confidence/credible interval), ideally using structured tables or plots.	Results
Results of syntheses	20a	For each synthesis, briefly summarise the characteristics and risk of bias among contributing studies.	Results
	20b	Present results of all statistical syntheses conducted. If meta-analysis was done, present for each the summary estimate and its precision (e.g. confidence/credible interval) and measures of statistical heterogeneity. If comparing groups, describe the direction of the effect.	Results
	20c	Present results of all investigations of possible causes of heterogeneity among study results.	Results
	20d	Present results of all sensitivity analyses conducted to assess the robustness of the synthesized results.	NA
Reporting biases	21	Present assessments of risk of bias due to missing results (arising from reporting biases) for each synthesis assessed.	NA
Certainty of evidence	22	Present assessments of certainty (or confidence) in the body of evidence for each outcome assessed.	Results
**DISCUSSION**			
Discussion	23a	Provide a general interpretation of the results in the context of other evidence.	Discussion
	23b	Discuss any limitations of the evidence included in the review.	Discussion
	23c	Discuss any limitations of the review processes used.	Discussion
	23d	Discuss implications of the results for practice, policy, and future research.	Discussion
**OTHER INFORMATION**			
Registration and protocol	24a	Provide registration information for the review, including register name and registration number, or state that the review was not registered.	NA

Section and Topic	Item #	Checklist item	Location where item is reported
	24b	Indicate where the review protocol can be accessed, or state that a protocol was not prepared.	NA
	24c	Describe and explain any amendments to information provided at registration or in the protocol.	Method
Support	25	Describe sources of financial or non-financial support for the review, and the role of the funders or sponsors in the review.	Discussion
Competing interests	26	Declare any competing interests of review authors.	Discussion
Availability of data, code and other materials	27	Report which of the following are publicly available and where they can be found: template data collection forms; data extracted from included studies; data used for all analyses; analytic code; any other materials used in the review.	Discussion

Supplementary 2. Data base search terms and search results

DB	Search	Search term	# of records
**PubMed**	#1	"RNA, Small Interfering"[Mesh]	75,961
	#2	"RNA, Small Interfering"[TW] OR "Interfering RNA, Small"[TW] OR "Small Interfering RNA"[TW] OR "Short Interfering RNA"[TW] OR "Interfering RNA, Short"[TW] OR "RNA, Short Interfering"[TW] OR "siRNA"[TW] OR "Short Hairpin RNA"[TW] OR "Hairpin RNA, Short"[TW] OR "RNA, Short Hairpin"[TW] OR "Small Hairpin RNA"[TW] OR "Hairpin RNA, Small"[TW] OR "RNA, Small Hairpin"[TW] OR "shRNA"[TW] OR "Scan RNA"[TW] OR "scnRNA"[TW] OR "Small Scan RNA"[TW] OR "RNA, Small Scan"[TW] OR "Scan RNA, Small"[TW] OR "RNA, Scan"[TW] OR "Trans-Acting siRNA"[TW] OR "Trans Acting siRNA"[TW] OR "tasiRNA"[TW] OR "siRNA, Trans-Acting"[TW] OR "siRNA, Trans Acting"[TW] OR "Repeat-Associated siRNA"[TW] OR "Repeat Associated siRNA"[TW] OR "siRNA, Repeat-Associated"[TW] OR "siRNA, Repeat Associated"[TW]	149,422
	#3	"RNA Interference"[Mesh]	57,313
	#4	"RNA Interference"[TW] OR "Interference, RNA"[TW] OR "RNAi"[TW] OR "Posttranscriptional Gene Silencing"[TW] OR "Gene Silencing, Posttranscriptional"[TW] OR "Gene Silencings, Posttranscriptional"[TW] OR "RNA Silencing"[TW] OR "Post-Transcriptional Gene Silencing"[TW] OR "Post Transcriptional Gene Silencing"[TW] OR "Post-Transcriptional Gene Silencings"[TW] OR "Silencing, Post-Transcriptional Gene"[TW] OR "Gene Silencing, Post-Transcriptional"[TW] OR "Gene Silencing, Post Transcriptional"[TW] OR "Cosuppression"[TW] OR "Co-Suppression"[TW] OR "Co Suppression"[TW]	96,474
	**#5 Combine**	**#1 OR #2 OR #3 OR #4**	**209,143**
	#6	"patisiran" [Supplementary Concept]	100
	#7	"patisiran"[TW] OR "Onpattro"[TW] OR "ALN-TTR02"[TW]	376
	#8	"lumasiran" [Supplementary Concept]	29
	#9	"lumasiran"[TW] OR "AD-65585"[TW] OR "ALN-G01"[TW] OR "ALN-65585"[TW] OR "oxlumo"[TW]	99
	#10	"ALN-PCS" [Supplementary Concept]	181
	#11	"ALN-PCS"[TW] OR "ALN-PCSsc"[TW] OR "inclisiran"[TW] OR "leqvio"[TW]	547
	#12	"givosiran" [Supplementary Concept]	44
	#13	"givosiran"[TW] OR "Givlaari"[TW]	121
	#14	"Angiotensinogen"[Mesh]	3,688
	#15	"Angiotensinogen"[TW] OR "Hypertensinogen"[TW] OR "Proangiotensin"[TW] OR "Renin-Substrate"[TW] OR "Renin Substrate"[TW] OR "Serpin A8"[TW] OR "SERPINA8"[TW] OR "Renin Substrate Tetradecapeptide"[TW] OR "Tetradecapeptide, Renin Substrate"[TW]	6,419
	#16	"Lipoprotein(a)"[Mesh]	6,412
	#17	"Lipoprotein(a)"[TW] OR "Lipoprotein a"[TW] OR "Lipoprotein Lp(a)"[TW] OR "Lipoprotein (a)"[TW] OR "Lipoprotein (a-)"[TW]	10,897
	#18	"vutrisiran"[TW] OR "SYL1001"[TW] OR "RG6346"[TW] OR "fazirsiran"[TW] OR "Cemdisiran"[TW] OR "ARC-AAT"[TW]	114
	#19	"nedosiran" [Supplementary Concept] OR "nedosiran"[TW]	39

	#20	"Hepatitis B, Chronic"[Mesh] OR "Hepatitis B Surface Antigens"[Mesh]	39,430
	#21	"Hemophilia A"[Mesh] OR "Hemophilia B"[Mesh]	25,924
	#22	"Angiopoietin-Like Protein 3"[Mesh]	490
	#23	"plozasiran"[TW] OR "zodasiran"[TW] OR "revusiran"[TW] OR "solbinsiran"[TW]	58
	**#24 Combine**	**#6 OR #7 OR #8 OR #9 OR #10 OR #11 OR #12 OR #13 OR #14 OR #15 OR #16 OR #17 OR #18 OR #19 OR #20 OR #21 OR #22 OR #23**	**84,153**
	**#25 Combine**	**#5 AND #24**	**1,369**

DB	Search	Search term	# of records
**EMBASE**	#1	"small interfering RNA"/exp	175,016
	#2	"RNA, Small Interfering":ti,ab,kw OR "Interfering RNA, Small":ti,ab,kw OR "Small Interfering RNA":ti,ab,kw OR "Short Interfering RNA":ti,ab,kw OR "Interfering RNA, Short":ti,ab,kw OR "RNA, Short Interfering":ti,ab,kw OR "siRNA":ti,ab,kw OR "Short Hairpin RNA":ti,ab,kw OR "Hairpin RNA, Short":ti,ab,kw OR "RNA, Short Hairpin":ti,ab,kw OR "Small Hairpin RNA":ti,ab,kw OR "Hairpin RNA, Small":ti,ab,kw OR "RNA, Small Hairpin":ti,ab,kw OR "shRNA":ti,ab,kw OR "Scan RNA":ti,ab,kw OR "scnRNA":ti,ab,kw OR "Small Scan RNA":ti,ab,kw OR "RNA, Small Scan":ti,ab,kw OR "Scan RNA, Small":ti,ab,kw OR "RNA, Scan":ti,ab,kw OR "Trans-Acting siRNA":ti,ab,kw OR "Trans Acting siRNA":ti,ab,kw OR "tasiRNA":ti,ab,kw OR "siRNA, Trans-Acting":ti,ab,kw OR "siRNA, Trans Acting":ti,ab,kw OR "Repeat-Associated siRNA":ti,ab,kw OR "Repeat Associated siRNA":ti,ab,kw OR "siRNA, Repeat-Associated":ti,ab,kw OR "siRNA, Repeat Associated":ti,ab,kw	175,771
	#3	"RNA interference"/exp	79,466
	#4	"RNA Interference":ti,ab,kw OR "Interference, RNA":ti,ab,kw OR "RNAi":ti,ab,kw OR "Posttranscriptional Gene Silencing":ti,ab,kw OR "Gene Silencing, Posttranscriptional":ti,ab,kw OR "Gene Silencings, Posttranscriptional":ti,ab,kw OR "RNA Silencing":ti,ab,kw OR "Post-Transcriptional Gene Silencing":ti,ab,kw OR "Post Transcriptional Gene Silencing":ti,ab,kw OR "Post-Transcriptional Gene Silencings":ti,ab,kw OR "Silencing, Post-Transcriptional Gene":ti,ab,kw OR "Gene Silencing, Post-Transcriptional":ti,ab,kw OR "Gene Silencing, Post Transcriptional":ti,ab,kw OR "Cosuppression":ti,ab,kw OR "Co-Suppression":ti,ab,kw OR "Co Suppression":ti,ab,kw	78,907
	**#5 Combine**	**#1 OR #2 OR #3 OR #4**	**304,925**
	#6	"patisiran"/exp	1,230
	#7	"patisiran":ti,ab,kw OR "Onpattro":ti,ab,kw OR "ALN-TTR02":ti,ab,kw	716
	#8	"lumasiran"/exp	325
	#9	"lumasiran":ti,ab,kw OR "AD-65585":ti,ab,kw OR "ALN-G01":ti,ab,kw OR "ALN-65585":ti,ab,kw OR "oxlumo":ti,ab,kw	188
	#10	"ALN-PCS":ti,ab,kw OR "leqvio":ti,ab,kw OR "ALN-PCSsc":ti,ab,kw OR "inclisiran":ti,ab,kw	900
	#11	"givosiran"/exp	395

	#12	"givosiran":ti,ab,kw OR "Givlaari":ti,ab,kw	224
	#13	"angiotensinogen"/exp	7,706
	#14	"Angiotensinogen":ti,ab,kw OR "Hypertensinogen":ti,ab,kw OR "Proangiotensin":ti,ab,kw OR "Renin-Substrate":ti,ab,kw OR "Renin Substrate":ti,ab,kw OR "Serpin A8":ti,ab,kw OR "SERPINA8":ti,ab,kw OR "Renin Substrate Tetradecapeptide":ti,ab,kw OR "Tetradecapeptide, Renin Substrate":ti,ab,kw	7,692
	#15	"lipoprotein A"/exp	13,818
	#16	"Lipoprotein(a)":ti,ab,kw OR "Lipoprotein a":ti,ab,kw OR "Lipoprotein Lp(a)":ti,ab,kw OR "Lipoprotein (a)":ti,ab,kw OR "Lipoprotein (a-)":ti,ab,kw	13,809
	#17	"vutrisiran":ti,ab,kw OR "SYL1001":ti,ab,kw OR "RG6346":ti,ab,kw OR "fazirsiran":ti,ab,kw OR "Cemdisiran":ti,ab,kw OR "ARC-AAT":ti,ab,kw	235
	#18	"nedosiran sodium"/exp OR "nedosiran":ti,ab,kw	122
	#19	"chronic hepatitis b"/exp OR "hepatitis b surface antigen"/exp	67,860
	#20	"hemophilia a"/exp OR "hemophilia b"/exp	36,761
	#21	"angiopoietin like protein 3"/exp	811
	#22	"plozasiran":ti,ab,kw OR "zodasiran":ti,ab,kw OR "revusiran":ti,ab,kw OR "solbinsiran":ti.ab.kw	94
	**#21 Combine**	**#6 OR #7 OR #8 OR #9 OR #10 OR #11 OR #12 OR #13 OR #14 OR #15 OR #16 OR #17 OR #18 OR #19 OR #20 OR #21 OR #22**	**135,395**
	**#22 Combine**	**#5 AND #21**	**3,955**

DB	Search	Search term	# of records
**Cochrane Library**	#1	[mh "RNA, Small Interfering"]	152
	#2	"RNA, Small Interfering":ti,ab,kw OR "Interfering RNA, Small":ti,ab,kw OR "Small Interfering RNA":ti,ab,kw OR "Short Interfering RNA":ti,ab,kw OR "Interfering RNA, Short":ti,ab,kw OR "RNA, Short Interfering":ti,ab,kw OR "siRNA":ti,ab,kw OR "Short Hairpin RNA":ti,ab,kw OR "Hairpin RNA, Short":ti,ab,kw OR "RNA, Short Hairpin":ti,ab,kw OR "Small Hairpin RNA":ti,ab,kw OR "Hairpin RNA, Small":ti,ab,kw OR "RNA, Small Hairpin":ti,ab,kw OR "shRNA":ti,ab,kw OR "Scan RNA":ti,ab,kw OR "scnRNA":ti,ab,kw OR "Small Scan RNA":ti,ab,kw OR "RNA, Small Scan":ti,ab,kw OR "Scan RNA, Small":ti,ab,kw OR "RNA, Scan":ti,ab,kw OR "Trans-Acting siRNA":ti,ab,kw OR "Trans Acting siRNA":ti,ab,kw OR "tasiRNA":ti,ab,kw OR "siRNA, Trans-Acting":ti,ab,kw OR "siRNA, Trans Acting":ti,ab,kw OR "Repeat-Associated siRNA":ti,ab,kw OR "Repeat Associated siRNA":ti,ab,kw OR "siRNA, Repeat-Associated":ti,ab,kw OR "siRNA, Repeat Associated":ti,ab,kw	447
	#3	[mh "RNA Interference"]	45

#4	"RNA Interference":ti,ab,kw OR "Interference, RNA":ti,ab,kw OR "RNAi":ti,ab,kw OR "Posttranscriptional Gene Silencing":ti,ab,kw OR "Gene Silencing, Posttranscriptional":ti,ab,kw OR "Gene Silencings, Posttranscriptional":ti,ab,kw OR "RNA Silencing":ti,ab,kw OR "Post-Transcriptional Gene Silencing":ti,ab,kw OR "Post Transcriptional Gene Silencing":ti,ab,kw OR "Post-Transcriptional Gene Silencings":ti,ab,kw OR "Silencing, Post-Transcriptional Gene":ti,ab,kw OR "Gene Silencing, Post-Transcriptional":ti,ab,kw OR "Gene Silencing, Post Transcriptional":ti,ab,kw OR "Cosuppression":ti,ab,kw OR "Co-Suppression":ti,ab,kw OR "Co Suppression":ti,ab,kw	372
**#5 Combine**	**#1 OR #2 OR #3 OR #4**	**726**
#6	"patisiran":ti,ab,kw OR "Onpattro":ti,ab,kw OR "ALN-TTR02":ti,ab,kw	142
#7	"lumasiran":ti,ab,kw OR "AD-65585":ti,ab,kw OR "ALN-G01":ti,ab,kw OR "ALN-65585":ti,ab,kw OR "oxlumo":ti,ab,kw	39
#8	"ALN-PCS":ti,ab,kw OR "leqvio":ti,ab,kw OR "ALN-PCSsc":ti,ab,kw OR "inclisiran":ti,ab,kw	156
#9	"givosiran":ti,ab,kw OR "Givlaari":ti,ab,kw	44
#10	[mh "Angiotensinogen"]	126
#11	"Angiotensinogen":ti,ab,kw OR "Hypertensinogen":ti,ab,kw OR "Proangiotensin":ti,ab,kw OR "Renin-Substrate":ti,ab,kw OR "Renin Substrate":ti,ab,kw OR "Serpin A8":ti,ab,kw OR "SERPINA8":ti,ab,kw OR "Renin Substrate Tetradecapeptide":ti,ab,kw OR "Tetradecapeptide, Renin Substrate":ti,ab,kw	325
#12	[mh "Lipoprotein(a)"]	565
#13	"Lipoprotein(a)":ti,ab,kw OR "Lipoprotein a":ti,ab,kw OR "Lipoprotein Lp(a)":ti,ab,kw OR "Lipoprotein (a)":ti,ab,kw OR "Lipoprotein (a-)":ti,ab,kw	1,715
#14	"vutrisiran":ti,ab,kw OR "SYL1001":ti,ab,kw OR "RG6346":ti,ab,kw OR "fazirsiran":ti,ab,kw OR "Cemdisiran":ti,ab,kw OR "ARC-AAT":ti,ab,kw	99
#15	"nedosiran":ti,ab,kw	7
#16	[mh "Hepatitis B, Chronic"] OR [mh "Hepatitis B Surface Antigens"]	2,339
#17	[mh "Hemophilia A"] OR [mh "Hemophilia B"]	683
#18	[mh "Angiopoietin-Like Protein 3"]	28
#19	"plozasiran":ti,ab,kw OR "zodasiran":ti,ab,kw OR "revusiran":ti,ab,kw OR "solbinsiran":ti,ab,kw	33
**#17 Combine**	**#6 OR #7 OR #8 OR #9 OR #10 OR #11 OR #12 OR #13 OR #14 OR #15 OR #16**	**5,559**
**#18 Combine**	**#5 AND #17**	**203**

Supplementary 3. McMaster Tool for Assessing Quality of Harms Assessments and Reporting in Study Reports (McHarm) of the included studies

**First author, Year [reference]**	Q1	Q2	Q3	Q4	Q5	Q6	Q7	Q8	Q9	Q10	Q11	Q12	Q13	Q14	Q15	McHarm Score*
Adams, 2018 [20]	Y	Y	Y	Y	Y	N	Y	N	Y	Y	Y	Y	Y	Y	Y	13
Coelho, 2013 [21]	Y	Y	Y	Y	Y	N	Y	N	Y	Y	Y	N	N	Y	Y	11
Frishberg, 2021 [22]	Y	Y	Y	Y	Y	N	Y	N	Y	Y	Y	Y	Y	Y	Y	13
Garrelf, 2021 [23]	Y	Y	Y	Y	Y	N	Y	N	Y	Y	Y	Y	Y	Y	Y	13
Balwani, 2020 [24]	Y	Y	Y	Y	Y	N	Y	N	Y	Y	Y	Y	Y	Y	Y	13
Maurer, 2023 [25]	Y	Y	Y	Y	Y	N	Y	N	Y	Y	Y	Y	Y	Y	Y	13
Raal, 2023 [26]	Y	Y	Y	Y	Y	N	Y	N	Y	Y	Y	Y	Y	Y	Y	13
Raal, 2020 [27]	Y	Y	Y	Y	Y	N	Y	Y	Y	Y	Y	Y	Y	Y	Y	14
Ray, 2017 [28]	Y	Y	Y	Y	Y	N	Y	N	Y	Y	Y	Y	Y	Y	Y	13
Ray, 2020 [29]	Y	Y	Y	Y	Y	N	Y	Y	Y	Y	Y	Y	Y	Y	Y	14
Sardh, 2019 [30]	Y	Y	Y	Y	Y	N	Y	N	Y	Y	Y	Y	Y	Y	Y	13
Thielmann, 2021 [31]	Y	Y	U	Y	Y	U	U	N	Y	Y	Y	Y	Y	Y	N	10
Huo, 2024 [32]	Y	Y	Y	Y	U	U	U	U	U	Y	Y	Y	Y	Y	Y	10
Yamashita, 2024 [33]	Y	Y	Y	Y	Y	N	Y	N	Y	Y	Y	Y	Y	Y	Y	13
Watts, 2025 [34]	Y	Y	Y	Y	Y	N	Y	N	Y	Y	Y	Y	Y	Y	Y	13
Luo, 2023 [35]	Y	Y	Y	Y	Y	N	U	U	Y	Y	Y	Y	Y	N	U	10
Ballantyne, 2024 [36]	Y	Y	Y	Y	Y	N	Y	Y	Y	Y	Y	Y	Y	Y	Y	14
Gaudet, 2024 [37]	Y	Y	Y	Y	Y	N	Y	Y	Y	Y	Y	Y	Y	Y	Y	14
Turner, 2018 [38]	Y	Y	U	Y	Y	N	Y	N	N	Y	Y	N	Y	Y	N	9
Barratt, 2024 [39]	Y	Y	Y	Y	U	U	Y	N	Y	Y	Y	Y	Y	Y	Y	12
Clark, 2024 [40]	Y	Y	Y	Y	Y	N	Y	N	Y	Y	Y	Y	Y	Y	Y	13
Nissen, 2023 [41]	Y	Y	Y	Y	Y	N	Y	N	Y	Y	Y	Y	N	Y	Y	12
Nissen, 2025 [42]	Y	Y	Y	Y	Y	N	Y	N	Y	Y	Y	Y	Y	Y	Y	13
Baum, 2022 [43]	Y	Y	Y	Y	Y	Y	Y	N	Y	Y	Y	Y	Y	Y	Y	14
Goldfarb, 2023 [44]	Y	Y	Y	Y	U	U	N	N	Y	Y	Y	Y	Y	Y	Y	11
Donoghue, 2022 [45]	Y	Y	Y	Y	Y	N	Y	N	Y	Y	Y	Y	Y	Y	Y	13
Gane, 2023 [46]	Y	Y	Y	Y	U	U	U	N	N	Y	Y	Y	Y	Y	N	9
Castilo, 2016 [47]	N	U	U	Y	Y	U	Y	N	Y	N	U	Y	Y	Y	Y	8
Fontana, 2025 [48]	Y	Y	Y	Y	Y	N	Y	N	Y	Y	Y	Y	Y	Y	Y	13
Nissen, 2024 [49]	Y	Y	Y	Y	Y	N	Y	N	Y	Y	Y	Y	Y	Y	Y	13
Nissen, 2024 [50]	Y	Y	Y	Y	Y	N	Y	N	Y	Y	Y	Y	Y	Y	Y	13
Bakris, 2024 [51]	Y	Y	Y	Y	Y	N	Y	N	Y	Y	Y	Y	Y	Y	Y	13

Desai, 2023 [52]	Y	Y	Y	Y	Y	N	Y	N	Y	Y	Y	Y	Y	Y	Y	13
Rosenson, 2024 [53]	Y	Y	Y	Y	Y	N	Y	N	Y	Y	Y	Y	Y	Y	Y	13
Judge, 2020 [54]	Y	Y	Y	Y	U	U	Y	N	Y	Y	Y	Y	Y	Y	Y	12
Desai, 2025 [55]	Y	Y	Y	Y	Y	N	Y	N	Y	Y	U	Y	Y	Y	Y	12
Koren, 2022 [56]	Y	N	N	Y	U	U	U	U	Y	Y	U	Y	Y	Y	Y	8
Nissen, 2022 [57]	Y	Y	Y	Y	Y	N	Y	N	Y	Y	U	Y	Y	Y	Y	12
Wiegman, 2025 [58]	Y	Y	Y	Y	Y	Y	Y	U	Y	Y	Y	Y	Y	Y	Y	14
Gottlieb, 2016 [59]	U	Y	Y	Y	U	U	N	N	Y	U	U	Y	Y	Y	N	7
Gane, 2023 [60]	U	Y	Y	Y	U	N	Y	N	Y	U	Y	N	Y	N	Y	8
Ray, 2025 [61]	Y	Y	Y	U	Y	N	Y	Y	Y	Y	Y	Y	Y	N	Y	12
Ray, 2025 [62]	Y	Y	Y	Y	Y	N	Y	Y	Y	Y	Y	Y	Y	Y	Y	14
Fabbrini, 2024 [63]	Y	Y	Y	Y	Y	U	Y	N	Y	Y	Y	N	Y	Y	Y	12
Zamora, 2011 [64]	U	Y	Y	Y	U	U	N	N	Y	U	Y	Y	Y	Y	N	8
Suzuki, 2017 [65]	U	U	Y	U	U	U	N	N	Y	N	Y	Y	Y	Y	Y	7
Yuen, 2020 [66]	U	Y	Y	Y	Y	U	Y	U	Y	N	Y	Y	Y	Y	N	10
Srivastava, 2023 [67]	Y	Y	Y	Y	Y	Y	Y	N	Y	Y	Y	Y	Y	Y	Y	14
Young, 2023 [68]	Y	Y	Y	Y	Y	Y	Y	N	Y	Y	Y	Y	Y	Y	Y	14
Hou, 2024 [69]	Y	Y	Y	Y	Y	Y	Y	N	Y	Y	Y	Y	Y	Y	Y	14
Doi, 2023 [70]	Y	Y	Y	Y	Y	Y	Y	N	Y	Y	Y	Y	Y	Y	Y	14
Koren, 2024 [71]	Y	Y	Y	Y	Y	Y	Y	N	Y	Y	Y	Y	Y	Y	Y	14
Taub, 2025 [72]	Y	Y	Y	Y	U	U	U	N	Y	U	Y	Y	Y	N	U	8

Y = Yes; U = Unable to determine; N = No; * = McHarm score defined as total count of Y in each article.
Q1: Were the harms PRE-DEFINED using standardized or precise definitions?
Q2: Were SERIOUS events precisely defined?
Q3: Were SEVERE events precisely defined?
Q4: Were the number of DEATHS in each study group specified OR were the reason(s) for not specifying them given?
Q5: Was the mode of harms collection specified as ACTIVE?
Q6: Was the mode of harms collection specified as PASSIVE?
Q7: Did the study specify WHO collected the harms?
Q8: Did the study specify the TRAINING or BACKGROUND of who ascertained the harms?
Q9: Did the study specify the TIMING and FREQUENCY of collection of the harms?
Q10: Did the author(s) use STANDARD scale(s) of checklist(s) for harms collection?
Q11: Did the authors specify if the harms reported encompass ALL the events collected or a selected SAMPLE?
Q12: Was the NUMBER of participants that withdrew or were lost to follow up specified for each study group?
Q13: Was the TOTAL NUMBER of participants affected by harms specified for each study arm?
Q14: Did the author(s) specify the NUMBER for each TYPE of harmful event for each study group?
Q15: Did the author(s) specify the type of analyses undertaken for harms data?

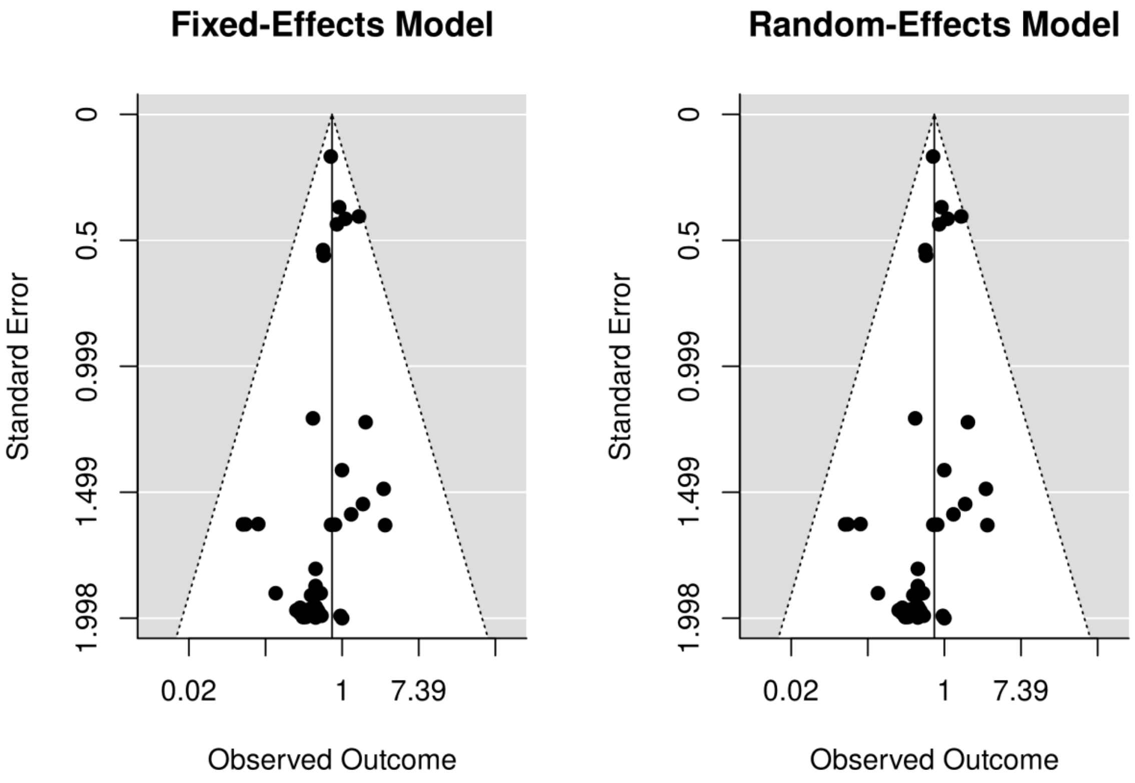

Supplementary 4. Funnel plot for publication bias for the included studies
